# Adaptive Use of Co‐Data Through Empirical Bayes for Bayesian Additive Regression Trees

**DOI:** 10.1002/sim.70004

**Published:** 2025-02-18

**Authors:** Jeroen M. Goedhart, Thomas Klausch, Jurriaan Janssen, Mark A. van de Wiel

**Affiliations:** ^1^ Department of Epidemiology & Data Science, Amsterdam Public Health Research Institute Amsterdam University Medical Centers Location AMC Noord Holland The Netherlands; ^2^ Department of Pathology, Cancer Center Amsterdam Amsterdam University Medical Centers Location VUMC Noord Holland The Netherlands

**Keywords:** Bayesian additive regression trees, co‐data, empirical Bayes, high‐dimensional data, omics, prediction

## Abstract

For clinical prediction applications, we are often faced with small sample size data compared to the number of covariates. Such data pose problems for variable selection and prediction, especially when the covariate‐response relationship is complicated. To address these challenges, we propose to incorporate external information on the covariates into Bayesian additive regression trees (BART), a sum‐of‐trees prediction model that utilizes priors on the tree parameters to prevent overfitting. To incorporate external information, an empirical Bayes (EB) framework is developed that estimates, assisted by a model, prior covariate weights in the BART model. The proposed EB framework enables the estimation of the other prior parameters of BART as well, rendering an appealing and computationally efficient alternative to cross‐validation. We show that the method finds relevant covariates and that it improves prediction compared to default BART in simulations. If the covariate‐response relationship is non‐linear, the method benefits from the flexibility of BART to outperform regression‐based learners. Finally, the benefit of incorporating external information is shown in an application to diffuse large B‐cell lymphoma prognosis based on clinical covariates, gene mutations, DNA translocations, and DNA copy number data.

## 1 Introduction

Modern statistical models for high‐dimensional data can often impose a prior weighting structure on the covariates to enhance variable selection and prediction. For instance, in penalized regression, such structure corresponds to how strong each covariate is regularized, and for tree‐based methods, the structure corresponds to the prior probabilities that covariates are selected in the splitting rules. Such weighting structures may be learned from external data sources. External data may be discrete such as a grouping of the covariates, or this data may be continuous such as estimated p‐values and effect size estimates of the covariates from a previous related study. We consider scenarios where such external data, from now on termed co‐data, is available.

Several linear models learn a covariate weighting structure from co‐data. Such models often utilize a Bayesian framework to naturally incorporate the co‐data through the priors, either by employing empirical or full Bayes. Most co‐data learners only handle discrete co‐data in the form of an *a priori* grouping of the covariates. Examples include GRridge [1], which employs empirical Bayes (EB), and Graper [2], which is full Bayes. Recently, linear models that allow multiple sources of co‐data, both continuous and discrete, were developed: Ecpc [3], which is EB‐based, and Fwen [4], which differs from the aforementioned methods by utilizing non‐Bayesian estimation to learn covariate weights.

For more flexible models such as tree ensembles, fewer co‐data methods are available, whereas these models may greatly benefit from prior weights to reduce the search in parameter space. One notable contribution is CoRF, which learns covariate weights from both discrete and continuous co‐data to enrich a random forest model [5]. Here, we develop an EB‐based method to incorporate discrete and continuous co‐data into Bayesian additive regression trees (BART), a prediction model that naturally allows for uncertainty quantification and that has been shown to often outperform other tree‐based methods [6].

BART is a sum‐of‐trees model, embedded in a Bayesian framework, which may estimate continuous and binary [6], and survival responses [7, 8, 9]. The main advantage of a sum‐of‐trees model is that additive and interaction effects are learned non‐parametrically from the primary data. To prevent overfitting, BART utilizes regularization priors that favor shallow trees and small response estimates. BART distinguishes itself from a random forest by mainly modeling low‐order interaction effects, whereas a random forest by default grows deeper trees and hence incorporates high‐order interaction effects. Therefore, BART may be considered more interpretable compared to a random forest.

BART has been extended to embed a sparsity structure in the covariate‐response relationship (DART) [10], possibly with an additional grouping structure of the covariates [11]. Both methods cannot handle non‐grouped or multi‐source co‐data. Furthermore, these methods are specifically designed for sparse settings, whereas high‐dimensional applications may often be rather dense, as argued for many omics settings [12].

We present EB‐coBART, a BART model that learns a weighting structure on the covariates by empirical Bayes, and a parsimonious co‐data model. This parsimony ensures stable estimated weights for the covariates. We show that our method adapts to the informativeness of the co‐data, so EB‐coBART hardly overfits in the case of non‐relevant co‐data.

Our method has several assets compared to aforementioned approaches to co‐data learning. First, EB‐coBART handles multi‐source co‐data, which may be both discrete and continuous. Second, our method accommodates both sparse and dense settings. Third, co‐data is naturally incorporated using Empirical Bayes (EB), in contrast to CoRF which relies on an ad hoc incorporation of the co‐data [5]. Furthermore, EB is easily implemented in existing BART software, whereas full Bayes approaches require rebuilding the algorithm from scratch.

Additionally, our derived EB framework may be used to estimate the other prior parameters of BART, thereby avoiding cross‐validation. Although we focus on estimating co‐data‐driven prior parameters, we also provide EB estimators for the other prior parameters and showcase them in an application.

This article is organized as follows. We review BART in Section 2. In Section 3, we first derive an empirical Bayes framework that may estimate any prior parameter of BART. Then, we describe how the co‐data model is included in this framework to estimate the covariate weights. Next, we present simulations in Section 4 and we illustrate our method in an application to lymphoma patients in Section 5. We conclude with a summary and a discussion in Section 6.

## 2 Summary of BART

Bayesian additive regression trees (BART) is a sum‐of‐trees model [6], which combines several weak learners, that is the individual trees, into a powerful model. Individual trees are discouraged from having a large effect on the sum by employing regularization priors that favor shallow trees and small terminal node values. Because our proposed method relies on several aspects of the original BART model, we present a detailed summary. We focus on regression, but BART and our proposed method also generalize to classification using a probit link and the augmentation strategy proposed by Albert and Chib [13].

### 2.1 A Sum‐Of‐Trees Model With Regularization Priors

Suppose we have a data set $D={\left\{\left(y_{i},x_{i}\right)\right\}}_{i=1}^{N}$ consisting of N observations of a normally distributed response $y_{i}$ and a p‐dimensional vector of covariates $x_{i}=\left(x_{1},\ldots ,x_{p}\right)$ with $x_{j}$ representing covariate j. We model $y_{i}$ by $y_{i}=f_{0}\left(x_{i}\right)+\epsilon_{i}$, with input $x_{i}$ and $\epsilon_{i}∼𝒩\left(0,\sigma^{2}\right).$ We then aim to estimate function $f_{0}\left(x_{i}\right).$

BART estimates $f_{0}\left(x_{i}\right)$ by a sum‐of‐trees model $f\left(x_{i};𝒯,ℳ\right)$ with parameters (𝒯,ℳ):

(1) $$f\left(x_{i};𝒯,ℳ\right)=\mu_{0}+\overset{K}{\underset{t=1}{\sum }}g\left(x_{i};{𝒯}_{t},{ℳ}_{t}\right)$$

with constant mean $\mu_{0}$ usually set to the average response $\bar{y}.$ In (1), g denotes a regression tree, having tree structure parameter ${𝒯}_{t}$ and terminal node parameter ${ℳ}_{t},$ and K denotes the number of trees, which we assume fixed (usually K=50 or K=200) [6]. Parameters 𝒯 and ℳ collect the K tree structures ${𝒯}_{t}$ and terminal node parameters ${ℳ}_{t},$ respectively.

In BART, (1) is embedded in a Bayesian framework with likelihood

(2) $$\pi \left(y|X,𝒯,ℳ,\sigma^{2}\right)=\overset{N}{\underset{i=1}{\prod }}𝒩\left(y_{i};f\left(x_{i};𝒯,ℳ\right),\sigma^{2}\right)$$

with $y={\left(y_{1},\ldots ,y_{N}\right)}^{T}$ and $X={\left(x_{1}^{T},\ldots ,x_{N}^{T}\right)}^{T},$ and priors π(𝒯,ℳ) and $\pi \left(\sigma^{2}\right).$ These priors have prior parameters ψ, which we specify in Section 2.2.

For binary responses, we model $p(y_{i}=1|x_{i})=\Phi \left(f\left(x_{i};𝒯,ℳ\right)\right),$ with constant $\mu_{0}=\Phi^{-1}\left(\bar{y}\right)$ and Φ the normal cdf with error variance $\sigma^{2}=1$. Therefore, prior $\pi \left(\sigma^{2}\right)$ is not required for binary response.

The posterior $\pi \left(𝒯,ℳ,\sigma^{2}|y,X;\psi \right),$ with given ψ, of the model parameters $\left(𝒯,ℳ,\sigma^{2}\right)$ is not analytical and hence a MCMC sampler is employed. We shortly summarize this sampler in Supporting Information S1: Section 1. Sampling details are described elsewhere [6].

We assess the convergence of MCMC samples by estimating the improved potential scale reduction factor R [14]. For continuous response, we consider the error variance $\sigma^{2}$ as convergence parameter and for binary response, we consider $f\left(x_{i};𝒯,ℳ\right)$ [15].

### 2.2 Prior Specification for BART

The error variance $\sigma^{2}$ has the standard prior $\pi \left(\sigma^{2};\nu ,\lambda \right)=ℐ𝒢\left(\sigma^{2};\frac{\nu }{2},\frac{\nu \lambda }{2}\right),$ with prior parameters ν and λ. The prior π(𝒯,ℳ) on the tree parameter is simplified by assuming that each tree t is i.i.d. with prior $\pi \left({𝒯}_{t},{ℳ}_{t}\right)=\pi \left({ℳ}_{t}|{𝒯}_{t}\right)\pi \left({𝒯}_{t}\right)$:

(3) $$\pi \left(𝒯,ℳ\right)=\overset{K}{\underset{t=1}{\prod }}\pi \left({ℳ}_{t}|{𝒯}_{t}\right)\pi \left({𝒯}_{t}\right)$$

with tree structure prior $\pi \left({𝒯}_{t}\right)$ and terminal node prior $\pi \left({ℳ}_{t}|{𝒯}_{t}\right).$

Priors $\pi \left({𝒯}_{t}\right)$ and $\pi \left({ℳ}_{t}|{𝒯}_{t}\right)$ are set such that each tree is expected to contribute slightly to the overall fit. This regularization is achieved by favoring shallow trees with small terminal node values. Before specifying these priors, we review the relevant parameters of a tree.

#### 2.2.1 Tree Parameterization

The tree structure ${𝒯}_{t}$ of tree t is parameterized by a set of nodes which are internal or terminal

(4) $${𝒯}_{t}=\left(\xi_{1t}(x_{j},a_{j},d),\ldots ,\xi_{Z_{t}t}(x_{j},a_{j},d),\omega_{1t}(d),\ldots ,\omega_{L_{t}t}(d)\right)$$

with $z=1,\ldots ,Z_{t}$ indexing the internal nodes $\xi_{zt},$ and $l=1,\ldots ,L_{t}$ indexing the terminal nodes $\omega_{lt}.$ An internal node ξ is parameterized by a binary splitting rule $\left\{x_{j}\leq a_{j}\right\},$ with $x_{j}$ the chosen splitting variable and $a_{j}$ a splitting value within the range of covariate j. Both the internal nodes and the terminal nodes have a depth parameter d, with the root node having d=0. We omitted the topological structure of the tree.

Terminal node $\omega_{lt}$ also has a parameter $\mu_{lt}$, which represents the response estimate for the given node. All $L_{t}$ estimates are collected in the terminal node parameter ${ℳ}_{t}=\left(\mu_{1t},\ldots ,\mu_{L_{t}t}\right)$ for tree t.

#### 2.2.2 Tree Structure Prior

The tree structure prior on ${𝒯}_{t}$ is chosen such that nodes have a probability of $\alpha {\left(1+d\right)}^{-\beta }$ to be internal and $1-\alpha {\left(1+d\right)}^{-\beta }$ to be terminal, with prior parameters α∈(0,1) and β>0. Prior parameters α and β are usually set at α=0.95 and β=2. A larger β and a smaller α favor shallower trees.

For internal nodes, splitting variables $x_{j}$ are chosen from a categorical prior with prespecified probabilities S for each j: $S=\left(s_{1},\ldots ,s_{p}\right).$ Default BART sets equal covariate weights: $s_{j}=1/p.$ In our proposed method, S will be estimated using Empirical Bayes and co‐data.

We have the following prior on tree structure ${𝒯}_{t}$:

(5) $$\begin{array}{ll} & \pi \left({𝒯}_{t};\alpha ,\beta ,S\right)\propto \left[\overset{Z_{t}}{\underset{z=1}{\prod }}\text{Categorical}\left(j_{zt};S\right)\right]\\ & \;\left[\overset{Z_{t}}{\underset{z=1}{\prod }}\alpha {\left(1+d_{zt}\right)}^{-\beta }\right]\left[\overset{L_{t}}{\underset{l=1}{\prod }}1-\alpha {\left(1+d_{lt}\right)}^{-\beta }\right] \end{array}$$

with $Z_{t}$ the number of internal nodes of tree t, $j_{zt}$ covariate j occurring in the splitting rule of the zth internal node of tree t, $L_{t}$ the number of terminal nodes, and $d_{zt}$ and $d_{lt}$ the depths of the zth internal node and the lth terminal node, respectively. Splitting values $a_{jz}$ are not considered in (5) because these values are assumed to be uniformly distributed.

#### 2.2.3 Terminal Node Prior

The $L_{t}$ terminal node parameters $\mu_{lt},$ collected in ${ℳ}_{t},$ of tree t are *a priori* assumed to be i.i.d. with a centered normal distribution:

(6) $$\pi \left({ℳ}_{t}|{𝒯}_{t};k\right)=\overset{L_{t}}{\underset{l=1}{\prod }}𝒩\left(\mu_{lt};0,\sigma_{\mu }^{2}\right),\;\sigma_{\mu }=\frac{0.5}{k\sqrt{K}}$$

with prior parameter k typically 1≤k≤3. Terminal node values are shrunken more for larger k or number of trees K. For binary responses, we have $\sigma_{\mu }=3/k\sqrt{K}.$

## 3 EB‐Cobart

It may be beneficial for BART to upweight (groups of) covariates that are expected to be informative for the response Y. In BART, this is naturally done by modifying prior parameter $S=\left(s_{1},\ldots ,s_{p}\right)$ of the categorical splitting variable prior, which normally defaults to a discrete uniform: $s_{j}=1/p$. Our main contribution is to impose a weighting structure in S in a data‐driven manner. To do so, we combine empirical Bayes (EB) with a co‐data model.

Empirical Bayes allows adaptively learning the structure in S with a central role for the primary data $D={\left\{\left(y_{i},x_{i}\right)\right\}}_{i=1}^{N}.$ However, estimating S by pure EB requires estimating a p‐dimensional prior parameter, which likely leads to overfitting for large p. Hence, we model the EB estimate of S by a co‐data model, which renders a substantial reduction in the dimension of the estimated prior parameter. Additionally, the co‐data model ensures a natural incorporation of external information on the covariates into BART.

We start by deriving EB for BART with the primary goal to estimate S. Our derived EB scheme for BART also renders estimators for the other prior parameters (α,β,k,ν,λ), thereby providing an alternative to cross‐validation if prior parameter tuning is desired. Next, we describe the co‐data model that guides the EB‐estimate of S. This completes our proposed methodology, which we call EB‐coBART. EB‐coBART is summarized in Algorithm 1. We end this section with remarks on choices for the other prior parameters.

### 3.1 Empirical Bayes for BART

We collect all prior parameters of BART in ψ=(α,β,k,ν,λ,S), which we then estimate by $\hat{\psi }$ using empirical Bayes (EB). To do so, we maximize the marginal likelihood ML(y|X;ψ) of BART w.r.t. $\psi :\;\hat{\psi }=\underset{\psi }{\arg\max}\;\text{ML}\left(y|X;\psi \right).$

The marginal likelihood of BART requires a summation over the full tree space [16, 17]. This exponentially large summation prevents direct maximization of ML(y|X;ψ). We therefore employ Casella's Monte Carlo EM algorithm [18], which maximizes ML(y|X;ψ) by iteratively updating prior parameters ψ using posterior samples of the model parameters.

Specifically, let $\theta =\left(𝒯,ℳ,\sigma^{2}\right)$ denote BART's model parameters and ${\hat{\psi }}^{\left(q\right)}$ the current (at iteration q) estimate of prior parameters ψ. Applying the EM algorithm then renders the next prior parameter estimates ${\hat{\psi }}^{\left(q+1\right)}$:

(7) $$\begin{array}{ll} {\hat{\psi }}^{\left(q+1\right)} & =\underset{\psi }{\arg\max}\;{𝔼}_{\pi \left(\theta |y,X;{\hat{\psi }}^{\left(q\right)}\right)}\left\{\log\left[\ell \left(\theta ,y|X;\psi \right)\right]\right\}\\ & =\underset{\psi }{\arg\max}\;{𝔼}_{\pi \left(\theta |y,X;{\hat{\psi }}^{\left(q\right)}\right)}\left\{\log\left[\pi \left(y|X,\theta \right)\pi \left(\theta ;\psi \right)\right]\right\} \end{array}$$

with ℓ(θ,y|X;ψ) the conditional likelihood of ψ [18], that is, the joint distribution of the data and the model parameters as a function of ψ, which we decompose by the product of the likelihood and the prior in the second line of (7).

Because (7) takes the expectation over the posterior of BART with prior parameters ${\hat{\psi }}^{\left(q\right)}$, (7) may be approximated by Monte Carlo integration using posterior MCMC samples $\theta_{m}^{\left(q\right)}=\left({𝒯}_{m}^{\left(q\right)},{ℳ}_{m}^{\left(q\right)},\sigma_{m}^{2(q)}\right)∼\pi \left(𝒯,ℳ,\sigma^{2}|y,X;{\hat{\psi }}^{\left(q\right)}\right),$ that is

(8) $${\hat{\psi }}^{\left(q+1\right)}\approx \underset{\psi }{\arg\max}\;\overset{n_{mc}}{\underset{m=1}{\sum }}\log\left[\pi \left({𝒯}_{m}^{\left(q\right)},{ℳ}_{m}^{\left(q\right)},\sigma_{m}^{2(q)};\psi \right)\right]$$

with m indexing posterior samples and $n_{mc}$ the total number of posterior samples. In (8), we dropped likelihood π(y|X,θ) because it does not depend on ψ. Note that $\pi \left({𝒯}_{m}^{\left(q\right)},{ℳ}_{m}^{\left(q\right)},\sigma_{m}^{2(q)};\psi \right)=\pi \left({𝒯}_{m}^{\left(q\right)},{ℳ}_{m}^{\left(q\right)};\alpha ,\beta ,k,S\right)\pi \left(\sigma_{m}^{2(q)}|{𝒯}_{m}^{\left(q\right)},{ℳ}_{m}^{\left(q\right)};\nu ,\lambda \right)$ evaluates the prior probabilities of the posterior samples as a function of ψ. The prior probabilities are evaluated using (3), (5), (6), and $\pi \left(\sigma_{m}^{2(q)}|{𝒯}_{m}^{\left(q\right)},{ℳ}_{m}^{\left(q\right)};\nu ,\lambda \right)=ℐ𝒢\left(\sigma_{m}^{2(q)};\frac{\nu }{2},\frac{\nu \lambda }{2}\right).$

Estimator (8) may be employed for all prior parameters (α,β,k,ν,λ,S). Here, we only show the EB‐estimator for prior covariate weights S, which has previously been shown to equal [19]:

(9) $${\hat{S}}_{1}^{\left(q+1\right)}=\left(b_{1}^{\left(q\right)}/B^{\left(q\right)},\ldots ,b_{p}^{\left(q\right)}/B^{\left(q\right)}\right)$$

with $b_{j}^{\left(q\right)}$ the number of splitting rules with covariate j at iteration q in the posterior sample of BART with prior parameter ${\hat{S}}_{1}^{\left(q1\right)},$ and $B^{\left(q\right)}$ the total number of sampled splitting rules. A derivation of (9) is found in Supporting Information S1: Section 2, as well as EB‐estimators for (α,β,k,ν,λ). Further details on our recommended choices for (α,β,k,ν,λ) are found in Section 3.4.

Estimator (9) of prior covariate weights S requires p parameter estimates. For large p, a scenario we are interested in, this estimator is unstable and likely overfits for the purpose of using it as a prior parameter in the next BART posterior sampling iteration. Therefore, we propose to smooth the feature‐specific estimates using co‐data, which substantially decreases the dimension of the estimator of S compared to (9). We denote this co‐data‐guided estimator by ${\hat{S}}_{2}^{\left(q+1\right)}.$

### 3.2 Co‐Data Model

Suppose we have κ co‐data variables of which we have complete measurements for each covariate j. We represent a grouping co‐data variable with G groups by dummy coding indicating which group covariate j belongs to. Covariates with missing co‐data observations may be defined as a separate group to account for potential information in the missingness [3].

We collect the co‐data measurements of each j, together with an intercept term, in the vector $c_{j}\in {\mathbb{R}}^{\kappa +1}$ and define the p×(κ+1) co‐data model matrix $𝒞={\left(c_{1}^{T},\ldots ,c_{p}^{T}\right)}^{T}.$ We then employ 𝒞 for modeling the feature‐specific EB estimates ${\hat{S}}_{1}^{\left(q+1\right)}$, that is, (9).

To link ${\hat{S}}_{1}^{\left(q+1\right)}$ to co‐data 𝒞, we model the counts $b_{j}^{\left(q\right)}.$ We start by noting that each of the in total $B^{\left(q\right)}$ splitting rules may be regarded as a Bernoulli trial for any covariate j: covariate j occurs or does not occur in the given splitting rule. Therefore, count $b_{j}^{\left(q\right)}$ is the outcome of a binomial trial: $b_{j}^{\left(q\right)}∼\mathrm{Bin}\left(w_{j}^{\left(q\right)},B^{\left(q\right)}\right),$ with unknown probability of success $w_{j}^{\left(q\right)}$ for covariate j at iteration q. We then model $w_{j}^{\left(q\right)}$ using 𝒞 by a logistic regression:

(10) $$w_{j}^{\left(q\right)}=\text{expit}\left(c_{j}^{T}\eta^{\left(q\right)}\right),\;\text{for}\;j=1,\ldots ,p$$

with $\text{expit}\left(x\right)=e^{x}/\left(e^{x}+1\right),$ and $\eta^{\left(q\right)}\in {\mathbb{R}}^{\kappa +1}$ a regression parameter vector.

We estimate $w_{j}^{\left(q\right)}$ by ${\hat{w}}_{j}^{\left(q\right)},$ which will be the co‐data moderated EB‐estimates ${\hat{S}}_{2}^{\left(q+1\right)}$ of prior parameter S. To determine ${\hat{w}}_{j}^{\left(q\right)},$ we first estimate $\eta^{\left(q\right)}$:

(11) $${\hat{\eta }}^{\left(q\right)}=\arg\underset{\eta^{\left(q\right)}}{\max}\;\overset{p}{\underset{j=1}{\sum }}\log\left[\mathrm{Bin}\left(b_{j}^{\left(q\right)};\;\text{expit}\left(c_{j}^{T}\eta^{\left(q\right)}\right),B^{\left(q\right)}\right)\right]$$

that is maximum likelihood maximization for logistic regression with covariates j acting as samples. Equation (11) ignores two dependencies between samples j. First, only one covariate can be used for a given splitting rule. Hence, success in a given Bernoulli's trial for variable j determines failure for the other variables. The second dependency originates from the dependence between the covariates, which induces dependencies between the splitting rules. Because we are only interested in the point estimates ${\hat{\eta }}^{\left(q\right)}$ and because the estimator (11) is consistent, we ignore these dependencies.

Estimate ${\hat{\eta }}^{\left(q\right)}$ then determines the estimates ${\hat{w}}_{j}^{\left(q\right)}=\text{expit}\left(c_{j}^{T}{\hat{\eta }}^{\left(q\right)}\right),$ which we collect in the co‐data guided empirical Bayes estimator ${\hat{S}}_{2}^{\left(q+1\right)}$ of S:

(12) $${\hat{S}}_{2}^{\left(q+1\right)}=\left({\hat{w}}_{1}^{\left(q\right)},\ldots ,{\hat{w}}_{p}^{\left(q\right)}\right)$$

Because the intercept term in ${\text{c}}_{j}$ ensures that the logistic regression model (10) is well‐calibrated, we have $\sum_{j=1}^{p}{\hat{w}}_{j}^{\left(q\right)}=\sum_{j=1}^{p}(b_{j}^{\left(q\right)}/B^{\left(q\right)})=1,$ that is, the sum of the predictions equals the sum of the responses. Therefore, (12) does not require re‐normalization.

Estimate ${\hat{\eta }}^{\left(q\right)}$ fully determines (12) given co‐data 𝒞. Hence, to determine ${\hat{S}}_{2}^{\left(q+1\right)}$, we only estimate κ+1 prior parameters instead of p, which is the case for (9). We therefore opt for a parsimonious model, that is, (10), with κ≪p and only linear effects.

Equation (10) ensures that the co‐data and the primary data agree on the informativeness of the co‐data. If, for example, the feature specific EB estimates of (9) show a strong preference for certain covariates, while this preference is not present in the co‐data, the final up/down weighting of the covariates will be diminished. Vice versa, if the co‐data shows a strong preference for a certain covariate, e.g., by a small p‐value, but BART does not recognize this covariate, the covariate will not be substantially upweighted.

### 3.3 Convergence of EB‐Cobart

Equation (12) provides iterative updates of prior parameter S and hence a convergence criterion is required. Typically, the marginal likelihood is tracked until stabilization [18]. However, for BART, estimating the marginal likelihood within reasonable computational time is non‐trivial. The harmonic mean estimator [20] requires too many samples to cover the tree space. Averaging the likelihood from prior sampling has been employed to estimate the marginal likelihood of BART [16], but only two covariates were considered. For larger p, the tree space becomes too large for this method to work. Chib proposed another option for marginal likelihood estimation [21]. This option is also unfeasible, because it requires an analytic expression of the full conditional of the tree structure parameter 𝒯 of BART, which is not available.

We therefore rely on the widely applicable information criterion (WAIC) [22]:

(13) $$\begin{array}{ll} \text{WAIC} & =-2\overset{N}{\underset{i=1}{\sum }}\log\left(E\left[\pi \left(y_{i}|𝒯,ℳ,\sigma^{2},x_{i}\right)\right]\right)\\ & \;+2\overset{N}{\underset{i=1}{\sum }}\mathrm{Var}\left[\log\left(\pi \left(y_{i}|𝒯,ℳ,\sigma^{2},x_{i}\right)\right)\right] \end{array}$$

with $\pi \left(y_{i}|𝒯,ℳ,\sigma^{2},x_{i}\right)$ the predictive density, and the expectation and variance taken w.r.t. the posterior of BART. We then halt the iterative prior parameter updates ${\hat{S}}_{2}^{\left(q+1\right)}$ when the WAIC is at minimum.

The WAIC as a convergence criterion has some desirable properties. First, the WAIC is easily computed for non‐parametric methods such as BART [23], contrary to other information criteria. Second, the WAIC is asymptotically equivalent to the leave‐one‐out cross‐validated likelihood.

As an alternative to the WAIC, the computationally more demanding cross‐validation (CV) may be used. In Supporting Information S1: Section 4, we illustrate in a simulation that CV as a stopping criterion leads to similar predictive performance and variable selection results compared to WAIC. Note that we also considered tracking prior parameter η until convergence but this led to overfitting (Supporting Information S1: Section 3).

ALGORITHM 1 **EB‐coBART algorithm to estimate prior parameter S.**

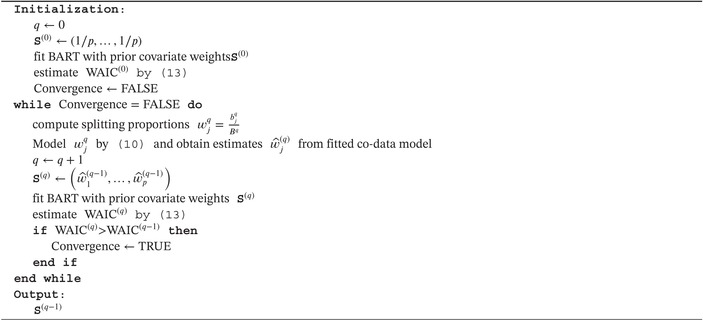

### 3.4 Choices for the Other Prior Parameters

Our derived EB scheme for BART also provides estimators for (α,β,k,ν,λ) (Supporting Information S1: Equations 12–15). Using EB for prior parameter estimation may be an appealing alternative to CV, which relies on a subjective grid of prior parameter choices.

Estimation of multiple (correlated) prior parameters, however, is intrinsically difficult. For example, for elastic net, a substantially less flexible prediction model compared to BART, it was proven that joint estimation of two prior parameters already causes identifiability issues [24].

Therefore, our default choice is to fix tree structure parameters α and β at two flexibility levels: rigid (α=0.1, β=4), favoring shallow trees and thus interpretability, and flexible (α=0.95, β=2), favoring less shallow trees. The flexible model corresponds to the default settings of BART [6]. Prior parameter k is fixed at k=1 for the rigid model and k=2 for the flexible model, acknowledging that shallow trees have more observations in the terminal nodes and thus require less shrinkage of the terminal node parameters. For the error variance prior parameters (ν,λ), we employ the default settings [6].

However, for high‐dimensional data, the predictions of BART may improve by prior parameter fine‐tuning [6]. In the high‐dimensional application (Section 5), we illustrate that hyperparameter estimation of α and k using the derived EB‐estimators renders a slight improvement in predictive performance compared to fixing α and k.

## 4 Simulations

We compare EB‐coBART to BART when informative co‐data is available in a high‐dimensional data setting. To do so, we consider two functions $f_{0}$ that specify a covariate‐response relationship: a sparse and non‐linear function (Section 4.1), and a dense and linear function (Section 4.2). For the sparse non‐linear function, we employ discrete co‐data in the form of a grouping structure, while for the dense linear function, we employ continuous co‐data in the form of a noisy version of the true linear effect sizes. Note that OG‐BART can handle discrete co‐data as well [11], but because its implementation is lacking in the public domain, we do not perform a comparison with this method. Section 4.3 deals with uninformative grouping co‐data to evaluate whether EB‐coBART recognizes such co‐data by not upweighting certain groups.

For both functions, we consider multiple simulation settings which are specified in the subsections. In each setting and for each function, we simulate $N_{\text{sim}}=500$ data sets. For each data set, we first fit BART, that is, BART with equal covariate weights $s_{j}=1/p.$ Then, we iteratively update $s_{j}$ according to estimator (12) until the WAIC, (13), is at minimum. BART fitted with updated $s_{j}$ then corresponds to EB‐coBART. To fit BART, we employ the R package dbarts [25], and to estimate the WAIC, we employ the R package loo [26].

We consider a rigid tree model (α=0.1, β=4, k=1), and a flexible tree model (α=0.95, β=2, k=2), as explained in Section 3.4. Thus, for each data set, we have four models: rigid BART, flexible BART, which is the default, rigid EB‐coBART, and flexible EB‐coBART.

We set the other prior parameter settings of the models as follows. We fix the error variance prior parameters ν=10 and λ such that the 75% quantile of the prior equals $2/3\hat{\mathrm{Var}}\left(y\right),$ with $\hat{\mathrm{Var}}\left(y\right)$ the estimated variance of the simulated response y. We also fix the number of trees to K=50 to balance prediction and variable selection [27].

For each simulated data set, we collect variable importance results by monitoring the co‐data‐moderated EB estimates ${\hat{w}}_{j}^{\left(q\right)},$ (12), which relate directly to how often the covariates occur in the tree ensemble. We estimate the predictive performance on a large ($N_{\text{test}}=500$) independent test set for both BART and EB‐coBART. We quantify the performance by the prediction mean square error (PMSE), that is, $\text{PMSE}=N_{\text{test}}^{-1}\sum_{i=1}^{N_{\text{test}}}{\left(y_{i}-{\hat{y}}_{i}\right)}^{2}$ and ${\hat{y}}_{i}$ denoting the prediction for sample i, which are obtained by averaging posterior samples of the sum‐of‐trees [6].

We compare the predictive performance of BART and EB‐coBART with several competitors. First, we consider DART [10], that is, BART with a sparsity‐inducing Dirichlet prior on $s_{j},$ which is implemented in the R package BART [15]. Second, we consider random forest [28], implemented in the R package randomForestSRC [29]. Last, we consider two other co‐data learners that can handle both discrete and continuous co‐data: Ecpc [3] implemented in the R package ecpc [30], which incorporates co‐data into ridge regression, and CoRF [5], which incorporates co‐data into a random forest model.

### 4.1 Sparse and Non‐Linear Setting

Response $y_{i}$ is generated by Friedman's five‐dimensional test function [31]: $y_{i}=f_{0}\left(x_{i}\right)+\epsilon_{i},$ with noise $\epsilon_{i}∼𝒩\left(0,1\right),$ for i=1,…,N, and with

(14) $$f_{0}\left(x_{i}\right)=10\sin\left(\pi x_{i1}x_{i2}\right)+10x_{i3}+20{\left(x_{i101}-0.5\right)}^{2}+10x_{i102}$$

and $x_{ij}\overset{i.i.d.}{˜}\text{Unif}\left(0,1\right),$ for j=1,…,p, and p=500. Thus, covariates {1,2,3,101,102} are predictive for the response and the remaining 495 covariates are noise.

Co‐data is defined as a grouping structure with G={5,20} groups. We set equal‐sized groups of size 100 for G=5 and size 25 for G=20. We then assign covariates 1,2,…,p/G to group 1, covariates p/G+1,…,2p/G to group 2, et cetera. Group G consists of covariates j=[(G−1)p/G+1],…,p. This distribution of covariates among the groups ensures that predictive covariates {1,2,3} are always in the same group (Group 1 for G=5 and G=20) and that predictive covariates {101,102} are always in the same group (Group 2 for G=5 and Group 5 for G=20). For both G's, we consider two sample sizes (N=100 and N=200).

**FIGURE 1:**
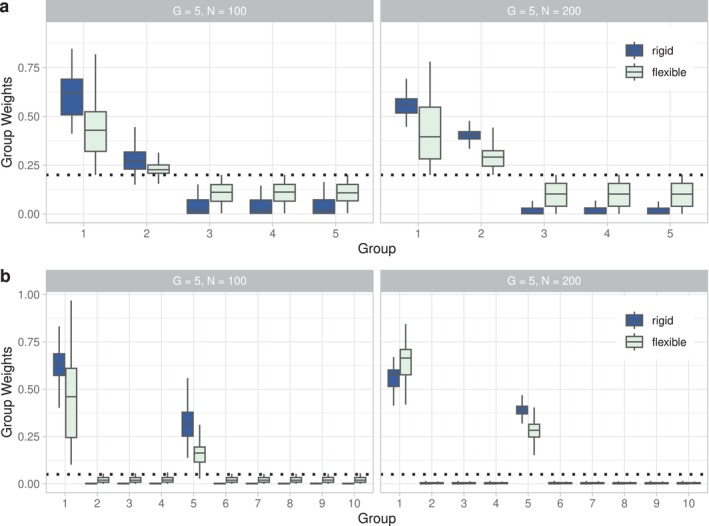
Boxplots of the co‐data moderated EB estimates of the covariate weights of EB‐coBART across the 500 simulated data sets for different simulation settings. Figure **(a)** shows results for G=5 and Figure **(b)** for G=20, for which we only depict the first 10 groups for visualization. For each group, the left (blue) boxplot corresponds to the rigid tree setting (α=0.1, β=4, k=1) and the right (green) boxplot to the flexible one (α=0.95, β=2, k=2). Outliers are not shown. The horizontal dotted lines correspond to equal group weights (0.2 for G=5; 0.05 for G=20).

Our results demonstrate that EB‐coBART upweights the predictive groups (1 and 2 for G=5; 1 and 5 for G=20) and downweights the non‐predictive groups in all simulation settings (Figure 1). This upweighting effect is stronger for the rigid tree models because shallow trees include fewer noisy, non‐relevant covariates in this sparse setting. Increasing the sample size from N=100 to N=200 reduces the variability in the group‐specific estimates across the data sets as expected. The upweighting of the predictive covariate groups by EB‐coBART also renders improved variable selection compared to BART (Supporting Information S1: Section 5 and Table S1).

For all simulation settings, EB‐coBART or DART has the smallest PMSE (Table 1). DART is expected to perform well because the simulated data is sparse and high‐dimensional. For G=5, DART has the best performance, while for G=20, the co‐data has become more informative and consequently EB‐coBART outperforms DART. EB‐coBART clearly outperforms co‐data learners Ecpc and CoRF. For Ecpc, we included a posthoc variable selection procedure based on the elastic net penalty [1, 3], using the optimal number of covariates $p_{\text{sel}}=5.$ Performances were worse with $p_{\text{sel}}=\{10,20\}.$

**TABLE 1:** Average PMSE across data sets for several simulation settings for BART and EB‐coBART in the rigid tree setting (α=0.1, β=4, k=1) and the flexible tree setting (α=0.95, β=2, k=2). Also included are competitors: DART, random forest, and co‐data learners: CoRF and Ecpc with and without post hoc variable selection.

	N=100,G=5	N=100,G=20	N=200,G=5	N=200,G=20
Flexible BART	11.4	11.4	4.58	4.58
Flexible EB‐coBART	10.1	7.63	4.23	3.23
Rigid BART	9.30	9.30	4.94	4.94
Rigid EB‐coBART	8.81	7.47	4.66	4.27
DART	8.24	**8.24**	4.04	4.04
Ecpc (no variable selection)	24.6	16.9	17.9	11.6
Ecpc (variable selection)	11.7	8.43	8.67	7.46
Random forest	20.7	20.7	15.4	15.4
CoRF	19.2	15.0	14.3	11.6

Rigid EB‐coBART has a lower PMSE than flexible EB‐coBART for N=100 and flexible EB‐coBART has a lower PMSE for N=200. In this sparse simulation setting, more regularization on the tree depth is required for smaller sample sizes.

**FIGURE 2:**
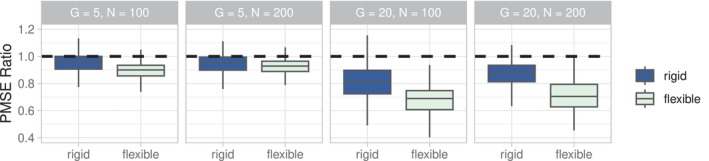
Boxplot of the ratio ${\text{PMSE}}_{\text{EBcoBART}}/{\text{PMSE}}_{\text{BART}}$ across the 500 simulated data sets for both the rigid tree models (blue, left) and the flexible tree models (green, right). The four panels correspond to different simulation settings.

Figure 2 compares the relative PMSE of EB‐coBART to that of BART demonstrating that EB‐coBART has a smaller PMSE than BART, that is, ${\text{PMSE}}_{\text{EBcoBART}}/{\text{PMSE}}_{\text{BART}}<1,$ for most data sets in all considered simulation settings and for both tree flexibility settings. Only for the rigid tree models in the G=5 setting, BART has a smaller PMSE than EB‐coBART for a relatively a large percentage of simulated data sets (19% for N=100; 22% for N=200).

Increasing the number of groups from G=5 to G=20 improves the relative performance of EB‐coBART compared to BART, because the co‐data has become relatively more informative. A larger sample size (N=200) renders a smaller relative difference in PMSE, that is, ${\text{PMSE}}_{\text{EBcoBART}}/{\text{PMSE}}_{\text{BART}}$ increases, compared to a smaller sample size (N=100). This finding is expected because prior information is more relevant for smaller sample sizes.

A comparison between the two tree flexibility settings reveals that the flexible tree models benefit more from the co‐data than the rigid tree models (Figure 2). The flexible model uses more nodes and can therefore relate better to the co‐data information. Moreover, the co‐data helps to prune away non‐informative covariates, which are less frequent for the rigid model.

### 4.2 Dense and Linear Setting

Details for the dense, linear setting with continuous co‐data are found in Supporting Information S1: Section 6. Briefly, EB‐coBART upweights predictive covariates and downweights non‐predictive covariates. This effect is, similar to the sparse non‐linear simulation, stronger for the rigid BART model (Supporting Information S1: Figure S5).

For most data sets, EB‐coBART has a lower PMSE than BART (Supporting Information S1: Figure S6). The flexible model has a lower average PMSE than the rigid model, because dense covariate‐response relationships require many splitting rules, which are more easily captured by a flexible model. The linear models Ecpc and ridge regression outperform all BART models because the data‐generating mechanism is linear. Among the non‐linear models, flexible EB‐coBART has the lowest average PMSE, and specifically a lower PMSE than DART, random forest, and CoRF (Supporting Information S1: Table S2).

### 4.3 Uninformative Co‐Data

EB‐coBART and BART perform similarly for uninformative co‐data. In the rigid tree setting, EB‐coBART does not upweight the groups on average, but there are fluctuations in the estimated group weights. These fluctuations also induce fluctuations in the test performance ratio, although on average both BART models have an almost equal PMSE. In the flexible tree setting, EB‐coBART and BART are practically identical. EB‐coBART does not upweight the groups on average and fluctuations in the estimated group weights across the data sets are hardly present. The PMSE of EB‐coBART and BART are close to equal for all data sets. All details are found in Supporting Information S1: Section 7.

## 5 Application

### 5.1 Description of the Data

We apply our method to the prognosis of Diffuse large B‐cell lymphoma (DLBCL) patients. DLBCL is a non‐Hodgkin lymphoma for which accurate prognosis is difficult because of the clinical and biological heterogeneity of the patients [32]. One well‐accepted prognostic clinical covariate is the international prognostic index (IPI) [33], which scores patients based on their age, the stage of the tumor, lactose dehydrogenase levels, mobility measure, and the number of extranodal sites. However, the predictive power of IPI is still limited. Therefore, a major branch of DLBCL research focuses on finding new omics‐based markers for DLBCL prognosis [32, 34].

To this end, we fit EB‐coBART to a cohort of 101 uniformly treated DLBCL patients for whom we aim to predict two‐year progression‐free survival (yes/no, 18% no). We treat the outcome as binary because two years is a clinically well‐accepted cut‐off and censoring was absent within this time period. We also have measurements of a total of p=140 covariates divided into four types: 67 DNA copy number variations (CNV) [32], 69 point mutations of genes, 3 translocations, and 1 clinical covariate: IPI.

We provide EB‐coBART with two co‐data sources. First, because we have different types of covariates which may have different scales in relation to the response, we group covariates by type (CNV, mutation, translocation, or clinical). IPI is a group on its own because of its known prognostic importance. Second, we provide continuous co‐data by estimating Benjamini‐Hochberg corrected p‐values [35] (−logit scale) of all covariates in association with the binary response from a combination of cohorts with N=430 DLBCL patients having received a slightly different treatment compared to the patients in the training cohort. In this way, EB‐coBART makes effective use of available data, while still acknowledging that the effect of prognostic factors may depend on treatment type. Because these cohorts have not been published yet, we provide anonymized data via https://github.com/JeroenGoedhart/EB_coBART_paper. Part of the co‐data cohort samples are publicly available [34].

### 5.2 Experimental Set‐Up

We fit EB‐coBART models to the training cohort. We consider EB‐coBART initialized in the flexible tree setting (α=0.95, β=2, k=2), which is the default [6], and EB‐coBART initialized in the rigid tree setting (α=0.95, β=2, k=2). For both tree settings, we consider two prior parameter estimation strategies. The first strategy (EB‐coBART 1) only estimates the covariate weight parameter S of BART by estimator (12), similar to Section 4, and the second strategy (EB‐coBART 2) simultaneously estimates S by (12) and prior parameters (α, k) according to (15) and (12) of the Supplements, with (α, k) initialized in the flexible or the rigid tree setting. Thus, we have four EB‐coBART models: flexible EB‐coBART 1, flexible EB‐coBART 2, rigid EB‐coBART 1, and rigid EB‐coBART 2. Prior parameter β is fixed to β=2 for flexible models and β=4 for the rigid models. We fit BART models using 10 chains each having 24 000 samples of which half is burn‐in.

We evaluate predictive performance by the area under the curve (AUC), estimated using R package pROC [36], and the average Brier score: $\text{Brier}=N_{\text{test}}^{-1}\sum_{i=1}^{N_{\text{test}}}{\left({\hat{y}}_{i}-y_{i}\right)}^{2}$. We estimate performances on an external test cohort consisting of 83 patients with the same treatment as patients in the training cohort. In addition, we investigate the predictive performance of EB‐coBART 2 as a function of the sample size by estimating a learning curve [37].

We compare EB‐coBART with flexible and rigid BART, that is, BART models having equal covariate weights ($s_{j}=1/p$), and with cv‐BART, which estimates prior parameters α, k, and the number of trees K using 5‐fold CV, and fixes β=2 and $s_{j}=1/p.$ We consider the CV grid α={0.1,0.5,0.95}, k={1,2,3}, and K={50,150}. We also fit BART using only IPI as a covariate (IPI‐BART). Last, we fit DART [10], BART with a sparsity‐inducing Dirichlet prior on $s_{j}$: $s_{j}∼\text{Dirichlet}(\tau /p,\ldots ,\tau /p)$ and τ/(τ+ρ)∼Beta(a,b). We show results for the default prior parameter settings [10]: a=1, b=0.5, and ρ=p. Tuning the sparsity by altering ρ did not improve performance. To fit DART, we employed the R package BART [15].

Next to the BART models, we include a comparison with random forest and its co‐data extension CoRF (both fitted using 2 000 trees with the R package randomForestSRC) [29], and to ridge regression and its co‐data extension Ecpc [3], both implemented in the R package ecpc [30]. For Ecpc, we also considered a posthoc variable selection with the optimal number of covariates $p_{\text{sel}}=\{2,5,10,50,80\}$, but this did not improve performance.

### 5.3 Results and Analysis

Predictive performance results are shown in Table 2. To facilitate comparison, and because the flexible tree setting (α=0.95, β=2, k=2) is the default [6], we show results for this setting here. Results for the rigid tree setting (α=0.1, β=4, k=1) (Supporting Information S1: Table S3) are summarized. Supporting Information S1: Table S3 also shows the performance evaluated internally on the training cohort, using repeated (3×) 10‐fold cross‐validation (CV).

**TABLE 2:** Predictive performance estimates, based on an external test cohort ($N_{\text{test}}=83$), of flexible BART, CV‐BART, that is, BART with prior parameter tuning by cross‐validation, EB‐coBART 1, and EB‐coBART 2, which estimates α and k by empirical Bayes and sets β=2. Also included are performance estimates of competitors: DART, random forest, CoRF, ridge regression, and Ecpc.

	AUC	Brier score
Flexible BART	0.557	0.162
CV‐BART	0.557	0.162
Flexible EB‐coBART 1	0.697	0.154
Flexible EB‐coBART 2	**0.714**	**0.153**
IPI‐BART	0.669	0.154
DART	0.552	0.163
Random Forest	0.629	0.158
CoRF	0.649	0.159
Ecpc	0.701	0.154
Ridge	0.693	0.154

Among the BART models, EB‐coBART 2 performs best, and slightly better than EB‐coBART 1. Because EB‐coBART 2 estimates α=0.61, it renders a sparser tree model compared to EB‐coBART 1, which uses α=0.95. The differences with BART, DART, and cv‐BART, which yields k=2, α=0.5, and K=150 as prior parameter estimates, are large. These differences are significant using DeLong's paired test for the difference in AUC ($p_{\Delta AUC}=0.013$, $p_{\Delta AUC}=0.017,$ and $p_{\Delta AUC}=0.024,$ respectively) [38], and the Wilcoxon signed rank test for the difference in Brier score ($p_{\Delta Brier}=0.034$, $p_{\Delta AUC}=0.017,$ and respectively).

**FIGURE 3:**
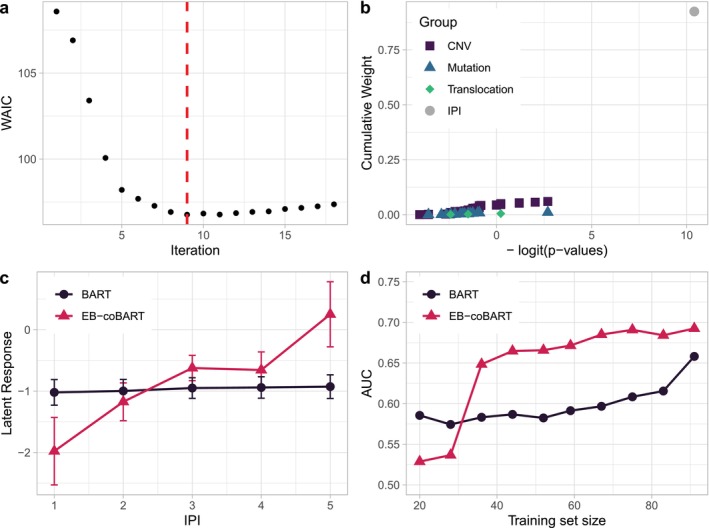
**(a)** Estimated WAIC (dots) for 18 iterations with the minimum indicated by the dashed vertical line at iteration 9. **(b)** Estimated cumulative covariate weights for the four types of covariates as a function of external p‐values on the −logit scale for EB‐coBART 2. Types of covariates are indicated by a square (copy number variation), a triangle (mutation), a diamond (translocation), and a circle (IPI). **(c)** Partial dependence plots of EB‐coBART 2 (triangles) and BART (dots) showing the marginal effect of IPI on the predictions. On the y‐axis, we show the latent response values, that is, Z‐values of the standard normal cdf, because BART models use a probit link for binary responses. We show the average ± of the standard deviation across the MCMC samples of the latent response. **(d)** Learning curves for EB‐coBART 2 (triangles) and BART (dots).

The superior performance of EB‐coBART compared to the other BART models may be explained by the estimated prior weight for IPI. The fit of EB‐coBART 2 is shown in Figure 3a (WAIC estimates across 18 iterations) and b (co‐data moderated EB estimates of the covariate weights ${\hat{w}}_{j}^{\left(q\right)}$, aggregated by covariate type, at minimum WAIC). At minimum WAIC (iteration 9), EB‐coBART 2 strongly upweights IPI, which receives 92.5% of the total weight. The remaining weight is spread out thinly among the omics covariates, with the sum of the weights per type equal to 6.00% for the copy number variations, 1.01% for the mutations, and 0.49% for the translocations. Devoting much weight to IPI suggests that EB‐coBART automatically finds a relevant predictive signal, because BART fitted with just IPI (IPI‐BART) has a substantially better performance than BART (Table 2). DART only shows a modest increase in the posterior splitting proportion of IPI (0.009 versus uniform weights 1/p=0.007). This suggests that employing co‐data renders a more predictive covariate structure on $s_{j}$ compared to assuming a sparsity structure on $s_{j}$.

The marginal effect of IPI on the predictions of EB‐coBART 2 and BART differ substantially. We quantify this marginal effect by the partial dependence function (Figure 3c) [39], and the average absolute Shapley value (Supporting Information S1: Figure S10) [40], which has been shown to accurately quantify variable importance for moderate‐to‐large p for BART [41]. Larger IPI values clearly decrease the probability of two‐year PFS for EB‐coBART 2 as expected (Figure 3d). For BART, this signal in IPI is absent. This finding is in agreement with the estimated difference in absolute average Shapley value of IPI for EB‐coBART and BART (Supporting Information S1: Figure S10).

The learning curves for flexible EB‐coBART 2 (triangles) and flexible BART (dots) are depicted in Figure 3d. Learning curves are created as follows. For ten different subsample sizes n, we sample multiple training sets without replacement from the training cohort and we define corresponding test sets as left‐out samples. We then fit flexible EB‐coBART 2 (updating $s_{j},$ α, k) and flexible BART to the training sets and estimate the AUC on the complementary test sets. We aggregate AUC estimates per subsample size by the average and plot them to obtain the learning curve.

For small subsample size n, BART has a larger AUC than EB‐coBART. Moving from n=28 to n=36, the AUC of EB‐coBART shows a phase transition by jumping to a much larger AUC value [42]. This transition is also present in the estimated weight for IPI, which makes a large jump at this transition subsample size (Supporting Information S1: Figure S9). The similar sample size trajectory of the predictive performance and the estimated prior weight on IPI of EB‐coBART again suggests the importance of IPI on the predictive power. The AUC of BART remains relatively constant across the sample size trajectory and only shows an increase in AUC at the end. The comparative trajectories of BART and EB‐coBART indicate that the inclusion of co‐data in the BART algorithm is most beneficial for medium sample sizes, as the sample size is then large enough to pick up the co‐data signal, while still too small to be picked up without co‐data guidance.

Rigid EB‐coBART and flexible EB‐coBART have similar performance (Supporting Information S1: Table S3), whereas rigid BART (without co‐data) performs better than flexible BART. This suggests a sparse covariate‐response relationship for this application, which, unlike for flexible BART, is picked up by flexible EB‐coBART as well due to the informative co‐data. Rigid EB‐coBART devotes slightly more prior weight to IPI (97%) compared to flexible EB‐coBART.

We end with a comparison between EB‐coBART and co‐data competitors CoRF and Ecpc (Table 2). EB‐coBART has a substantially, but not significantly, better test performance than random forest and CoRF, while Ecpc and ridge regression are competitive. Ecpc and CoRF benefit only marginally from the co‐data, indicated by the comparative performance of their corresponding base learners. In Supporting Information S1: Section 9, we show an application with a continuous outcome in which EB‐coBART clearly outperforms Ecpc (PMSE=0.378 versus PMSE=0.508), likely because of a non‐linear relationship.

## 6 Discussion

We developed EB‐coBART, a method that incorporates co‐data into BART by estimating prior covariate weights using empirical Bayes and a co‐data model. This method rendered improved predictions, depending on the informativeness of the co‐data, compared to standard BART in simulations and in an application to lymphoma patients. Furthermore, this application illustrated that EB‐coBART performs better than CoRF [5] and competitively to Ecpc [3], two state‐of‐the‐art co‐data learners. The comparative performance of EB‐coBART and regression‐based Ecpc depends on how well the true model can be approximated by a linear one.

The application illustrated that having co‐data‐informed prior covariate weights assists in separating signal covariates (IPI) from noisy covariates (omics‐based markers), which was not feasible without co‐data. The co‐data weights also enable researchers to evaluate the relative strength of different covariate types/groups, which may be more insightful than comparing individual covariates. Furthermore, the degree of prior up/down weighting of covariates informs research on how informative the co‐data is for the application at hand.

We considered EB‐coBART having a flexible tree setting, which is the default [6], and a rigid tree setting. The rigid model is sparser because it uses less nodes and it therefore lends itself better for variable selection and model interpretability compared to the flexible model. For prediction, we therefore recommend the rigid model when the performance is comparable, as was the case in the application.

EB‐coBART has to refit BART at each iteration and it is therefore computationally intensive. However, because we empirically found that 4–12 iterations is typical, EB‐coBART requires less computational time than cross‐validated BART. The computational time of BART, and thus also of EB‐coBART, does not scale well to very high‐dimensional settings ($p\approx 10^{4}$). It may therefore be interesting to apply our method to more computationally efficient BART‐based methods such as BART‐BMA or XBART [17, 43]. However, incorporation of empirical Bayes into these methods is not straightforward because formal EB requires a prior distribution on the splitting variables which is lacking for both BART‐BMA and XBART.

We end with suggestions for future research. First, for tree‐based methods, it is generally difficult to deal with types of covariates that have a different scale and priority in relation to the response. Grouping the covariates by type provided a simple solution, but other, more sophisticated, solutions may improve the tree‐based model. It may be good to favor certain types, for example, clinical covariates, at the trunk of the trees, as such covariates may be easier to interpret and may have proven their use more extensively. Second, EB‐coBART may be combined with Thompson sampling, a variable selection technique that was recently incorporated into BART [44]. This technique may benefit from weighting the covariates *a priori*, especially for complex high‐dimensional settings.

## Conflicts of Interest

The authors declare no conflicts of interest.

## Supporting information




**Data S1.** Supporting Information.

## Data Availability

For the DLBCL application, treatment and covariate names were anonymized, as the original data has not been published yet. Anonymized data include the training cohort, the test cohort, and the co‐data matrix. These data and R code (version 4.3.0) to reproduce results presented in Sections 4 and 5 are available via https://github.com/JeroenGoedhart/EB_coBART_paper. An R package implementing EBcoBART is available on CRAN via https://cran.r‐project.org/web/packages/EBcoBART/index.html. The package, which is called EBcoBART, also contains the anonymized data.

## References

[1] M. A. van de Wiel , T. G. Lien , W. Verlaat , W. N. van Wieringen , and S. M. Wilting , “Better Prediction by Use of co‐Data: Adaptive Group‐Regularized Ridge Regression,” Statistics in Medicine 35, no. 3 (2016): 368–381, 10.1002/sim.6732. 26365903

[2] B. Velten and W. Huber , “Adaptive Penalization in High‐Dimensional Regression and Classification With External Covariates Using Variational Bayes,” Biostatistics 22, no. 2 (2019): 348–364, 10.1093/biostatistics/kxz034. PMC8036004 31596468

[3] M. M. van Nee , L. F. A. Wessels , and M. A. van de Wiel , “Flexible co‐Data Learning for High‐Dimensional Prediction,” Statistics in Medicine 40, no. 26 (2021): 5910–5925, 10.1002/sim.9162. 34438466 PMC9292202

[4] J. Tay , N. Aghaeepour , T. Hastie , and R. Tibshirani , “Feature‐Weighted Elastic Net: Using “Features of Features” for Better Prediction,” Statistica Sinica 33 (2023): 259–279, 10.5705/ss.202020.0226. 37102071 PMC10129060

[5] D. E. te Beest , S. W. Mes , S. M. Wilting , R. H. Brakenhoff , and M. A. van de Wiel , “Improved High‐Dimensional Prediction With Random Forests by the Use of co‐Data,” BMC Bioinformatics 18, no. 1 (2017): 584, 10.1186/s12859-017-1993-1. 29281963 PMC5745983

[6] H. A. Chipman , E. I. George , and R. E. McCulloch , “BART: Bayesian Additive Regression Trees,” Annals of Applied Statistics 4, no. 1 (2010): 266–298, 10.1214/09-AOAS285.

[7] V. Bonato , V. Baladandayuthapani , B. M. Broom , E. P. Sulman , K. D. Aldape , and K. Do , “Bayesian Ensemble Methods for Survival Prediction in Gene Expression Data,” Bioinformatics 27, no. 3 (2010): 359–367, 10.1093/bioinformatics/btq660. 21148161 PMC3031034

[8] R. A. Sparapani , B. R. Logan , R. E. McCulloch , and P. W. Laud , “Nonparametric Survival Analysis Using Bayesian Additive Regression Trees (BART),” Statistics in Medicine 35, no. 16 (2016): 2741–2753, 10.1002/sim.6893. 26854022 PMC4899272

[9] R. A. Sparapani , B. R. Logan , M. J. Maiers , P. W. Laud , and R. E. McCulloch , “Nonparametric Failure Time: Time‐To‐Event Machine Learning With Heteroskedastic Bayesian Additive Regression Trees and Low Information Omnibus Dirichlet Process Mixtures,” Biometrics 79, no. 4 (2023): 3023–3037, 10.1111/biom.13857. 36932826 PMC10505620

[10] A. R. Linero , “Bayesian Regression Trees for High‐Dimensional Prediction and Variable Selection,” Journal of the American Statistical Association 113, no. 522 (2018): 626–636, 10.1080/01621459.2016.1264957.

[11] J. Du and A. R. Linero , “Incorporating Grouping Information Into Bayesian Decision Tree Ensembles,” Proceedings of Machine Learning Research 97 (2019): 1686–1695.

[12] E. A. Boyle , Y. I. Li , and J. K. Pritchard , “An Expanded View of Complex Traits: From Polygenic to Omnigenic,” Cell 169, no. 7 (2017): 1177–1186, 10.1016/j.cell.2017.05.038. 28622505 PMC5536862

[13] J. H. Albert and S. Chib , “Bayesian Analysis of Binary and Polychotomous Response Data,” Journal of the American Statistical Association 88, no. 422 (1993): 669–679, 10.1080/01621459.1993.10476321.

[14] A. Vehtari , A. Gelman , D. Simpson , B. C , and P. C. Bürkner , “Rank‐Normalization, Folding, and Localization: An Improved Rhat for Assessing Convergence of MCMC (With Discussion),” Bayesian Analysis 16, no. 2 (2021): 667–718, 10.1214/20-BA1221.

[15] R. A. Sparapani , C. Spanbauer , and R. E. McCulloch , “Nonparametric Machine Learning and Efficient Computation With Bayesian Additive Regression Trees: The BART R Package,” Journal of Statistical Software 97, no. 1 (2021): 1–66, 10.18637/jss.v097.i01.

[16] J. A. Boatman , D. M. Vock , and J. Koopmeiners , “Borrowing From Supplemental Sources to Estimate Causal Effects From a Primary Data Source,” Statistics in Medicine 40, no. 24 (2021): 5115–5130, 10.1002/sim.9114. 34155662 PMC11667477

[17] B. Hernández , A. E. Raftery , S. R. Pennington , and A. C. Parnell , “Bayesian Additive Regression Trees Using Bayesian Model Averaging,” Statistics and Computing 28, no. 4 (2018): 869–890, 10.1007/s11222-017-9767-1. 30449953 PMC6238959

[18] G. Casella , “Empirical Bayes Gibbs Sampling,” Biostatistics 2, no. 4 (2001): 485–500, 10.1093/biostatistics/2.4.485. 12933638

[19] A. R. Linero and J. Du , “Variable Selection for Bayesian Decision Tree Ensembles,” in Handbook of Bayesian Variable Selection, eds. G. M. Tadesse and M. Vannucci (New York: Chapman and Hall/CRC, 2021), 415–440.

[20] M. A. Newton and A. E. Raftery , “Approximate Bayesian Inference With the Weighted Likelihood Bootstrap,” Journal of the Royal Statistical Society, Series B: Statistical Methodology 56, no. 1 (1994): 3–48.

[21] S. Chib , “Marginal Likelihood From the Gibbs Output,” Journal of the American Statistical Association 90, no. 432 (1995): 1313–1321, 10.1080/01621459.1995.10476635.

[22] S. Watanabe , “A Widely Applicable Bayesian Information Criterion,” Journal of Machine Learning Research 14, no. 1 (2013): 867–897.

[23] J. S. Murray , “Log‐Linear Bayesian Additive Regression Trees for Multinomial Logistic and Count Regression Models,” Journal of the American Statistical Association 116, no. 534 (2021): 756–769, 10.1080/01621459.2020.1813587.

[24] M. M. van Nee , T. van de Brug , and M. A. van de Wiel , “Fast Marginal Likelihood Estimation of Penalties for Group‐Adaptive Elastic Net,” Journal of Computational and Graphical Statistics 32, no. 3 (2022): 950–960, 10.1080/10618600.2022.2128809. 38013849 PMC10511031

[25] V. Dorie , *“dbarts*: *Discrete Bayesian Additive Regression Trees Sampler*,” 2023 R package version 0.9–23.

[26] A. Vehtari , A. Gelman , and J. Gabry , “Practical Bayesian Model Evaluation Using Leave‐One‐Out Cross‐Validation and WAIC,” Statistics and Computing 27 (2017): 1413–1432, 10.1007/s11222-016-9696-4.

[27] J. Bleich , A. Kapelner , E. I. George , and S. T. Jensen , “Variable Selection for BART: An Application to Gene Regulation,” Annals of Applied Statistics 8, no. 3 (2014): 1750–1781, 10.1214/14-aoas755.

[28] L. Breiman , “Random Forests,” Machine Learning 45 (2001): 5–32, 10.1023/A:1010950718922.

[29] H. Ishwaran and U. B. Kogalur , “ *Fast* Unified Random Forests for Survival, Regression, and Classification (RF‐SRC),” 2023 R package version 3.2.2.

[30] M. M. van Nee , L. F. A. Wessels , and M. A. van de Wiel , “Ecpc an R‐Package for Generic co‐Data Models for High‐Dimensional Prediction,” BMC Bioinformatics 24, no. 1 (2023): 172, 10.1186/s12859-023-05289-x. 37101151 PMC10134536

[31] J. H. Friedman , “Multivariate Adaptive Regression Splines,” Annals of Statistics 19, no. 1 (1991): 1–67, 10.1214/aos/1176347963.

[32] M. Mendeville , J. Janssen , Y. Kim , v. E. Dijk , D. Jong , and B. Ylstra , “A Bioinformatics Perspective on Molecular Classification of Diffuse Large B‐Cell Lymphoma,” Leukemia 36, no. 9 (2022): 2177–2179, 10.1038/s41375-022-01670-6. 35933522 PMC9417979

[33] M. A. Shipp , D. P. Harrington , M. M. Klatt , et al., “Identification of Major Prognostic Subgroups of Patients With Large‐Cell Lymphoma Treated With m‐BACOD or M‐BACOD,” Annals of Internal Medicine 104, no. 6 (1986): 757–765, 10.7326/0003-4819-104-6-757. 2422995

[34] B. Chapuy , C. Stewart , A. J. Dunford , et al., “Molecular Subtypes of Diffuse Large B Cell Lymphoma Are Associated With Distinct Pathogenic Mechanisms and Outcomes,” Nature Medicine 24, no. 5 (2018): 679–690, 10.1038/s41591-018-0016-8. PMC6613387 29713087

[35] Y. Benjamini and Y. Hochberg , “Controlling the False Discovery Rate: A Practical and Powerful Approach to Multiple Testing,” Journal of the Royal Statistical Society: Series B: Methodological 57, no. 1 (1995): 289–300, 10.1111/j.2517-6161.1995.tb02031.x.

[36] X. Robin , N. Turck , A. Hainard , et al., “pROC: An Open‐Source Package for R and S+ to Analyze and Compare ROC Curves,” BMC Bioinformatics 12, no. 1 (2011): 77, 10.1186/1471-2105-12-77. 21414208 PMC3068975

[37] J. M. Goedhart , T. Klausch , and M. A. van de Wiel , “Estimation of Predictive Performance in High‐Dimensional Data Settings Using Learning Curves,” Computational Statistics and Data Analysis 180 (2023): 107622, 10.1016/j.csda.2022.107622.

[38] E. R. DeLong , D. M. DeLong , and D. L. Clarke‐Pearson , “Comparing the Areas Under Two or More Correlated Receiver Operating Characteristic Curves: A Nonparametric Approach,” Biometrics 44, no. 3 (1988): 837–845, 10.2307/2531595. 3203132

[39] J. H. Friedman , “Greedy Function Approximation: A Gradient Boosting Machine,” Annals of Statistics 29, no. 5 (2001): 1189–1232, 10.1214/aos/1013203451.

[40] L. S. Shapley , “A Value for n‐Person Games,” in Contributions to the Theory of Games II, eds. H. W. Kuhn and A. W. Tucker (Princeton: Princeton University Press, 1953), 307–317.

[41] A. Horiguchi and M. T. Pratola , “Estimating Shapley Effects for Moderate‐To‐Large Input Dimensions,” 2023. *ArXiv*.

[42] D. L. Donoho and J. Tanner , “Sparse Nonnegative Solution of Underdetermined Linear Equations by Linear Programming,” Proceedings of the National Academy of Sciences 102, no. 27 (2005): 9446–9451, 10.1073/pnas.0502269102. PMC1172251 15976026

[43] J. He , S. Yalov , and P. R. Hahn , “XBART: Accelerated Bayesian Additive Regression Trees,” Proceedings of Machine Learning Research 89 (2019): 1130–1138.

[44] Y. Liu and V. Ročková , “Variable Selection via Thompson Sampling,” Journal of the American Statistical Association 118, no. 541 (2023): 287–304, 10.1080/01621459.2021.1928514. PMC10281711 37346228

