# Supplementary material for: Adaptive Use of Co‐Data Through Empirical Bayes for Bayesian Additive Regression Trees
Source: Stat Med. 2025 Feb 18;44(5):e70004. doi: 10.1002/sim.70004 (PMC11834989; doi:10.1002/sim.70004)
Supplement: Supplementary file 1 — Data S1. Supporting Information. [file SIM-44-0-s001.pdf]

SUPPLEMENTARY MATERIAL TO: Adaptive Use of Co-data through Empirical Bayes for Bayesian Additive Regression Trees

Jeroen M. Goedhart*[1], Thomas Klausch[1], Jurriaan Janssen[2], Mark A. van de Wiel[1]

*Correspondence e-mail address: j.m.goedhart@amsterdamumc.nl

[1]Department of Epidemiology & Data Science, Amsterdam Public Health Research Institute, Amsterdam University Medical Centers Location AMC, Meibergdreef 9, the Netherlands

[2]Department of Pathology, Cancer Center Amsterdam, Amsterdam University Medical Centers Location VUMC, De Boelelaan 1117, the Netherlands

1 Sampling from the posterior of BART

The posterior of BART is proportional to

$$\begin{aligned}\pi\left(\boldsymbol{\mathcal{T}}, \boldsymbol{\mathcal{M}}, \sigma^2 \mid \boldsymbol{y}, \boldsymbol{X}; \alpha, \beta, k, \nu, \lambda, \boldsymbol{S}\right) \propto & \left[\prod_{i=1}^{N} \mathcal{N}\left(Y_i; G\left(\boldsymbol{x}_i; \boldsymbol{\mathcal{T}}, \boldsymbol{\mathcal{M}}\right), \sigma^2\right)\right] \\ & \times \left[\prod_{t=1}^{K}\prod_{l=1}^{L_t} \pi\left(\mathcal{T}_t; \alpha, \beta, \boldsymbol{S}\right) \mathcal{N}\left(\mu_{lt}; 0, \frac{0.5}{k\sqrt{K}}\right)\right] \\ & \times \left[\mathcal{IG}\left(\sigma^2; \frac{\nu}{2}, \frac{\nu\lambda}{2}\right)\right].\end{aligned} \tag{1}$$

To sample from the posterior, (1) is first decomposed into two full posterior conditionals:

$$\begin{aligned}\pi\left(\boldsymbol{\mathcal{T}}, \boldsymbol{\mathcal{M}} \mid \boldsymbol{y}, \boldsymbol{X}, \sigma^2; \alpha, \beta, k, \boldsymbol{S}\right) &= \pi\left(\boldsymbol{\mathcal{T}}, \boldsymbol{\mathcal{M}} \mid \boldsymbol{y}, \boldsymbol{X}; \alpha, \beta, k, \boldsymbol{S}\right) \\ \pi\left(\sigma^2 \mid \boldsymbol{\mathcal{T}}, \boldsymbol{\mathcal{M}}, \boldsymbol{y}, \boldsymbol{X}; \nu, \lambda\right) &= \mathcal{IG}\left(\sigma^2; \frac{N+\nu}{2}, \frac{\nu\lambda + \sum_{i=1}^{N}\left(y_i - \hat{y}_i\right)^2}{2}\right)\end{aligned} \tag{2}$$

The last line of (2) originates from the conjugacy of the inverse gamma prior. The first line of (2) requires further decomposition.

This decomposition is performed using a Bayesian backfitting algorithm proposed by Hastie and Tibshirani (2000). By noting that a tree t depends on all other trees only through the residual response $\boldsymbol{r}_t = \boldsymbol{y} - \sum_{t' \neq t}^{K-1} g\left(\boldsymbol{X}; \mathcal{T}_t, \mathcal{M}_{t'}\right)$, we may simplify a draw from the first line of (2) to K

draws of subsequent trees. A single tree Monte Carlo sample consists of first sampling a new tree structure $\mathcal{T}_t$ and then an update of the terminal node parameters $\mathcal{M}_t$:

$$\begin{aligned} \pi\left(\mathcal{T}_t \mid \boldsymbol{r}_t, \sigma^2; \alpha, \beta\right) &\propto \pi\left(\mathcal{T}_t; \alpha, \beta\right) p\left(\boldsymbol{r}_t \mid \mathcal{T}_t, \sigma^2\right) \\ \pi\left(\mathcal{M}_t \mid \mathcal{T}_t, \boldsymbol{r}_t, \sigma^2; k\right) &= \prod_{l=1}^{L_t} \pi\left(\mu_{lt} \mid \mathcal{T}_t, \boldsymbol{r}_{lt}, \sigma^2; k\right). \end{aligned} \tag{3}$$

A Metropolis-Hasting algorithm is employed to sample new tree structures $\mathcal{T}_t$ (first line of (3)). Here, a local tree modification proposal distribution is used for which full details are described by Tan and Roy (2019) and Kapelner and Bleich (2016). The conjugacy of the terminal node prior (eq. 2.6 of main text) ensures that the second line of (3) is a $\mathcal{N}\left(\mu_{lt}; \frac{\sigma^{-2}\sum_{h=1}^{n_{lt}} r_{hlt}}{n_{lt}/\left(\sigma^2+\sigma_\mu^2\right)}, \frac{1}{n_{lt}/\left(\sigma^2+\sigma_\mu^2\right)}\right)$, with n_{lt} the number of observations falling in the lth terminal node of tree t, and h an index variable representing the hth observation of a given terminal node and tree.

2 Stochastic EM algorithm

Here, we derive the stochastic EM algorithm for marginal likelihood maximization w.r.t. the hyperparameters for BART. Let $\boldsymbol{\psi}$ be a vector of hyperparameters and $\mathrm{ML}\left(\boldsymbol{y} \mid \boldsymbol{X}; \boldsymbol{\psi}\right)$ the marginal likelihood as a function of $\boldsymbol{\psi}$. In Empirical Bayes, the goal is to find the hyperparameters that satisfy

$$\hat{\boldsymbol{\psi}} = \underset{\boldsymbol{\psi}}{\arg\max}\ \mathrm{ML}\left(\boldsymbol{y} \mid \boldsymbol{X}; \boldsymbol{\psi}\right). \tag{4}$$

To approximate the optimization in (4), Casella (2001) proposed a stochastic EM algorithm that iteratively uses Gibbs samples from the posterior $\pi_{\boldsymbol{\psi}}\left(\boldsymbol{\theta} \mid \boldsymbol{y}, \boldsymbol{X}\right)$. This algorithm reads in general form

$$\hat{\boldsymbol{\psi}}^{(q+1)} = \underset{\boldsymbol{\psi}}{\arg\max}\ E_{\pi\left(\boldsymbol{\theta} \mid \boldsymbol{y}, \boldsymbol{X}; \hat{\boldsymbol{\psi}}^{(q)}\right)}\left[\log \ell\left(\boldsymbol{\theta}, \boldsymbol{y} \mid \boldsymbol{X}; \boldsymbol{\psi}\right)\right] \approx \underset{\boldsymbol{\psi}}{\arg\max}\ \frac{1}{n_{mc}} \sum_{m=1}^{n_{mc}} \log \ell\left(\boldsymbol{\theta}_m^{(q)}, \boldsymbol{y} \mid \boldsymbol{X}; \boldsymbol{\psi}\right),$$

with q the iteration index and $\ell\left(\boldsymbol{\theta}, \boldsymbol{y} \mid \boldsymbol{X}; \boldsymbol{\psi}\right)$ the conditional likelihood of $\boldsymbol{\psi}$, i.e. it evaluates the joint probability of data $\boldsymbol{y} \mid \boldsymbol{X}$ and model parameters $\boldsymbol{\theta}$ as a function of $\boldsymbol{\psi}$. The expectation of this conditional likelihood is taken w.r.t to the posterior distribution with hyperparameter $\boldsymbol{\psi}^{(q)}$. Then, for the right-hand side, we have m the mth Monte Carlo sample, n_{mc} the total number of

Monte Carlo samples, q the qth iteration, and $\ell\left(\boldsymbol{\theta}_m^{(q)}, \boldsymbol{y} \mid \boldsymbol{X}; \boldsymbol{\psi}\right)$ the conditional likelihood of $\boldsymbol{\psi}$ evaluated at the mth posterior sample of the model parameters $\boldsymbol{\theta}_m^{(q)}$. Specifically, $\boldsymbol{\theta}_m^{(q)}$ denotes the mth model parameter sample from the posterior $\pi\left(\boldsymbol{\theta} \mid \boldsymbol{y}, \boldsymbol{X}; \hat{\boldsymbol{\psi}}^{(q)}\right)$ with the hyperparameters set to $\hat{\boldsymbol{\psi}}^{(q)}$.

For BART, the model parameters are $\boldsymbol{\theta} = (\boldsymbol{\mathcal{T}}, \boldsymbol{\mathcal{M}}, \sigma)$, and the hyperparameters are $\boldsymbol{\psi} = (\alpha, \beta, k, \nu, \lambda, \boldsymbol{S})$, which leads to

$$\hat{\boldsymbol{\psi}}^{(q+1)} \approx \arg\max_{\boldsymbol{\psi}} \frac{1}{n_{mc}} \sum_{m=1}^{n_{mc}} \log \ell\left(\boldsymbol{y}, \boldsymbol{\mathcal{T}}_m^{(q)}, \boldsymbol{\mathcal{M}}_m^{(q)}, \sigma_m^{2(q)} \mid \boldsymbol{X}; \boldsymbol{\psi}\right), \tag{5}$$

with $\left(\boldsymbol{\mathcal{T}}_m^{(q)}, \boldsymbol{\mathcal{M}}_m^{(q)}, \sigma_m^{2(q)}\right)$ denoting the mth posterior sample of the tree parameters and the error variance at iteration q. By factoring out the joint likelihood and dropping all terms that do not depend on the hyperparameters, (5) can be rewritten as

$$\begin{aligned}\hat{\boldsymbol{\psi}}^{(q+1)} &\approx \arg\max_{\alpha,\beta,k,\nu,\lambda,\boldsymbol{S}} \frac{1}{n_{mc}} \sum_{m=1}^{n_{mc}} \log\left[\pi\left(\boldsymbol{y} \mid \boldsymbol{X}, \boldsymbol{\mathcal{T}}_m^{(q)}, \boldsymbol{\mathcal{M}}_m^{(q)}, \sigma_m^{2(q)}\right) \pi\left(\boldsymbol{\mathcal{T}}_m^{(q)}, \boldsymbol{\mathcal{M}}_m^{(q)}, \sigma_m^{2(q)}; \alpha, \beta, k, \nu, \lambda, \boldsymbol{S}\right)\right] \quad (6)\\ &= \arg\max_{\alpha,\beta,k,\nu,\lambda,\boldsymbol{S}} \sum_{m=1}^{n_{mc}} \log\left[\pi\left(\boldsymbol{\mathcal{T}}_m^{(q)}, \boldsymbol{\mathcal{M}}_m^{(q)}, \sigma_m^{2(q)}; \alpha, \beta, k, \nu, \lambda, \boldsymbol{S}\right)\right],\end{aligned}$$

with $\pi\left(\boldsymbol{\mathcal{T}}_m^{(q)}, \boldsymbol{\mathcal{M}}_m^{(q)}, \sigma_m^{2(q)}; \alpha, \beta, k, \nu, \lambda, \boldsymbol{S}\right)$ the prior probabilities of the mth posterior sample $\left(\boldsymbol{\mathcal{T}}_m^{(q)}, \boldsymbol{\mathcal{M}}_m^{(q)}, \sigma_m^{2(q)}\right)$, with hyperparameters $\hat{\boldsymbol{\psi}}^{(q)}$. Equation 6 is further decomposed as

$$\hat{\boldsymbol{\psi}}^{(q+1)} = \arg\max_{\alpha,\beta,k,\nu,\lambda,\boldsymbol{S}} \sum_{m=1}^{n_{mc}} \left[\log \pi\left(\boldsymbol{\mathcal{T}}_m^{(q)}; \alpha, \beta, \boldsymbol{S}\right) + \log \pi\left(\boldsymbol{\mathcal{M}}_m^{(q)} \mid \boldsymbol{\mathcal{T}}_m^{(q)}; k\right) + \log \pi\left(\sigma_m^{2(q)} \mid \boldsymbol{\mathcal{T}}_m^{(q)}, \boldsymbol{\mathcal{M}}_m^{(q)}; \nu, \lambda\right)\right],$$

thus rendering

$$\left(\hat{\alpha}^{(q+1)}, \hat{\beta}^{(q+1)}, \hat{\boldsymbol{S}}^{(q+1)}\right) = \underset{\alpha,\beta,\boldsymbol{S}}{\arg\max} \sum_{m=1}^{n_{mc}} \log \pi\left(\boldsymbol{\mathcal{T}}_m^{(q)}; \alpha, \beta, \boldsymbol{S}\right) \tag{7}$$

$$\begin{aligned}\hat{k}^{(q+1)} &= \underset{k}{\arg\max} \sum_{m=1}^{n_{mc}} \log \pi\left(\boldsymbol{\mathcal{M}}_m^{(q)} \mid \boldsymbol{\mathcal{T}}_m^{(q)}; k\right) \\ &= \underset{k}{\arg\max} \sum_{m=1}^{n_{mc}} \log\left(\prod_{t=1}^{K}\prod_{l=1}^{L_{tm}^{(q)}} \mathcal{N}\left(\mu_{ltm}^{(q)}; 0, \sigma_\mu^2\right)\right) \\ &= \underset{k}{\arg\max} \sum_{m=1}^{n_{mc}}\sum_{t=1}^{K}\sum_{l=1}^{L_{tm}^{(q)}} \log\mathcal{N}\left(\mu_{ltm}^{(q)}; 0, \frac{3}{k\sqrt{K}}\right)\end{aligned} \tag{8}$$

$$\left(\hat{\nu}^{(q+1)}, \hat{\lambda}^{(q+1)}\right) = \underset{\nu,\lambda}{\arg\max} \sum_{m=1}^{n_{mc}} \log \mathcal{IG}\left(\sigma_m^{2(q)}; \frac{\nu}{2}, \frac{\nu\lambda}{2}\right), \tag{9}$$

with $L_{tm}^{(q)}$ the number of terminal nodes of tree t in Monte Carlo sample m at iteration q, and $\mu_{ltm}^{(q)}$ the lth sampled terminal node of tree t in the mth Monte Carlo sample at iteration q. Now, (8) and (9) are standard maximum likelihood estimation problems for the normal and inverse gamma distribution, respectively. Equation 7 requires

$$\begin{aligned}\left(\hat{\alpha}^{(q+1)}, \hat{\beta}^{(q+1)}, \hat{\boldsymbol{S}}^{(q+1)}\right) &= \underset{\alpha,\beta,\boldsymbol{S}}{\arg\max} \sum_{m=1}^{n_{mc}} \log \prod_{t=1}^{K}\left[\prod_{z=1}^{Z_{tm}^{(q)}} \text{Categorical}\left(j_{ztm}^{(q)}; \boldsymbol{S}\right) \prod_{z=1}^{Z_{tm}^{(q)}} \alpha\left(1+d_{ztm}\right)^{-\beta} \prod_{l=1}^{L_{tm}^{(q)}} 1-\alpha\left(1+d_{ltm}\right)^{-\beta}\right], \\ &= \underset{\boldsymbol{S}}{\arg\max} \sum_{m=1}^{n_{mc}}\sum_{t=1}^{K} \log\left[\prod_{z=1}^{Z_{tm}^{(q)}} \text{Categorical}\left(j_{ztm}^{(q)}; \boldsymbol{S}\right)\right] \\ &\quad + \underset{\alpha,\beta}{\arg\max} \sum_{m=1}^{n_{mc}}\sum_{t=1}^{K} \log\left[\prod_{z=1}^{Z_{tm}^{(q)}} \alpha\left(1+d_{ztm}^{(q)}\right)^{-\beta} \prod_{l=1}^{L_{tm}^{(q)}} 1-\alpha\left(1+d_{ltm}^{(q)}\right)^{-\beta}\right]\end{aligned}$$

which renders the updates

$$\hat{\boldsymbol{S}}^{(q+1)} = \underset{\boldsymbol{S}}{\arg\max} \sum_{m=1}^{n_{mc}}\sum_{t=1}^{K}\sum_{z=1}^{Z_{tm}^{(q)}} \log\left[\text{Categorical}\left(j_{ztm}^{(q)}; \boldsymbol{S}\right)\right] \tag{10}$$

$$\left(\hat{\alpha}^{(q+1)}, \hat{\beta}^{(q+1)}\right) = \underset{\alpha,\beta}{\arg\max} \sum_{m=1}^{n_{mc}}\sum_{t=1}^{K} \log\left[\prod_{z=1}^{Z_{tm}^{(q)}} \alpha\left(1+d_{ztm}^{(q)}\right)^{-\beta} \prod_{l=1}^{L_{tm}^{(q)}} 1-\alpha\left(1+d_{ltm}^{(q)}\right)^{-\beta}\right], \tag{11}$$

with j_{ztm} denoting splitting variable x_j of the zth internal node of tree t and Monte Carlo sample m. Equation 10 is recognized as maximum likelihood estimation of a categorical distribution.

Collecting all results then leads to the following iterative EB updates

$$\hat{k}^{(q+1)} = \frac{\sum_{m=1}^{n_{mc}} \sum_{t=1}^{K} L_{tm}^{(q)}}{3\sqrt{\sum_{m=1}^{n_{mc}} \sum_{t=1}^{K} \sum_{l=1}^{L_{tm}^{(q)}} \left(\mu_{ltm}^{(q)}\right)^2} \sqrt{K}}, \tag{12}$$

$$\hat{\boldsymbol{S}}^{(q+1)} = \left(b_1^{(q)}/B^{(q)}, \dots, b_p^{(q)}/B^{(q)}\right), \tag{13}$$

$$\left(\hat{\nu}^{(q+1)}, \hat{\lambda}^{(q+1)}\right) = \underset{\nu,\lambda}{\arg\max} \sum_{m=1}^{n_{mc}} \log \mathcal{IG}\left(\sigma_m^{2(q)}; \frac{\nu}{2}, \frac{\nu\lambda}{2}\right), \tag{14}$$

$$\left(\hat{\alpha}^{(q+1)}, \hat{\beta}^{(q+1)}\right) = \underset{\alpha,\beta}{\arg\max} \sum_{m=1}^{n_{mc}} \sum_{t=1}^{K} \left[\sum_{z=1}^{Z_{tm}^{(q)}} \log\left(\alpha\left(1 + d_{ztm}^{(q)}\right)^{-\beta}\right) + \sum_{l=1}^{L_{tm}^{(q)}} \log\left(1 - \alpha\left(1 + d_{ltm}^{(q)}\right)^{-\beta}\right)\right], \tag{15}$$

with $b_j^{(q)}/B^{(q)}$ the estimated probability of covariate j getting selected in the splitting rules. Here, $b_j^{(q)}$ represents the total count of splitting rules with j at iteration q for all Monte Carlo samples combined and $B^{(q)}$ represents the total count of all splitting rules at iteration q.

Updates $\hat{k}^{(q+1)}$ and $\hat{\boldsymbol{S}}^{(q+1)}$ are calculated analytically, whereas updates $\left(\hat{\nu}^{(q+1)}, \hat{\lambda}^{(q+1)}\right)$ correspond to standard maximum likelihood estimation of an inverse gamma distribution. Updates $\left(\hat{\alpha}^{(q+1)}, \hat{\beta}^{(q+1)}\right)$ may be solved using the base R optim function.

3 Overfitting of EB updates

Here, we illustrate that it is unfeasible to use the estimated hyperparameters as stopping criterion for the stochastic EM algorithm. Typically, the hyperparameters are updated using EM until they stabilize within a given tolerance level. However, for BART, we empirically found that this stopping criterion is unfeasible to halt the EM algorithm of EB-coBART.

We first show overfitting of the co-data moderated EB-estimates of $\boldsymbol{S}$ by plotting the average PMSE, with the average taken over the 500 simulated data sets, as a function of the iteration number for the simulations described in the main text (Section 4). We remind the reader that at each iteration hyperparameter $\boldsymbol{S}$ is updated by eq. 3.12 of the main text. Figure S1 shows these results for the sparse and nonlinear simulation (Subsection 4.1 of main text) and Figure S2 shows results for the dense and linear simulation (Subsection 4.2 of main text and supplementary section 6). The simulation settings are specified in the different panels of Figure S1 and Figure S2.

Figure S1 and Figure S2 illustrate that the average test performance first decreases and then increases again, which hints to overfitting. This effect is stronger for the flexible tree models than for the rigid tree models. For the rigid tree models, the average performance stabilizes as a function of the iteration number in the sparse and nonlinear simulations.

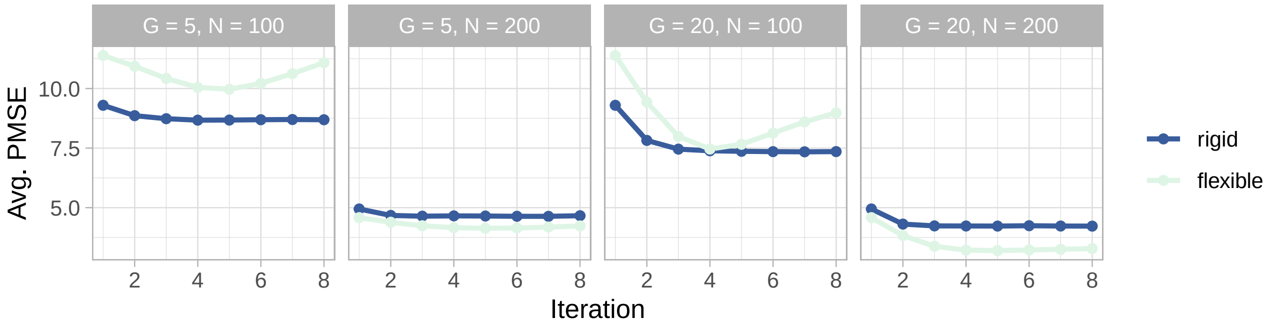

Figure S1: Average (over data sets) prediction mean square error (PMSE) as a function of the iteration number for the rigid tree setting (blue) and the flexible tree setting (green) for the sparse and nonlinear simulation with discrete co-data. The four different panels correspond to different simulation settings.

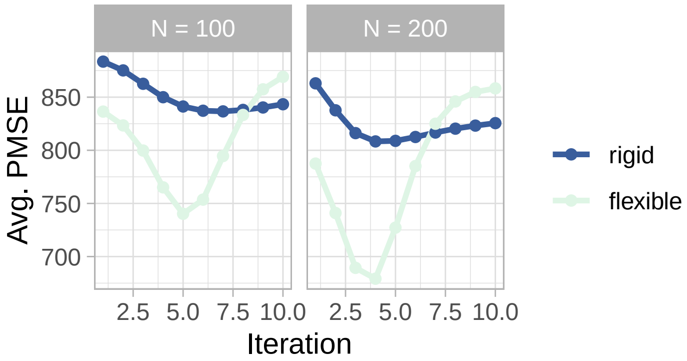

Figure S2: Average (over data sets) prediction mean square error (PMSE) as a function of the iteration number for the rigid tree setting (blue) and the flexible tree setting (green) for the linear and dense simulation with continuous co-data. The left panel corresponds to the $N = 100$ simulation setting and the right panel to the $N = 200$ setting.

Next, we show the co-data moderated EB estimates of S at several iteration numbers in the sparse and nonlinear setting. Specifically, we simulate $N_{sim} = 100$ data sets according to: $y_i = f(\boldsymbol{x}_i) + \epsilon, \quad \text{with} \quad \epsilon \sim \mathcal{N}(0,1), \quad \text{for} \quad i = 1, \ldots, N$, and with

$$f(\boldsymbol{x}_i) = 10\sin(\pi x_{i1} x_{i2}) + 20(x_{i101} - 0.5)^2 + 10x_{i3} + 10x_{i102}, \tag{16}$$

and $X_{ij} \sim \text{Unif}(0,1)$, for $j = 1, \ldots, p$ and $p = 500$. Thus, covariates $\{1, 2, 3, 101, 102\}$ are predictive for the response and the remaining 495 covariates are noise.

Co-data is defined as a grouping structure with $G = 5$ groups. We set equal-sized groups of size 100. We then assign covariates $1, 2, \ldots, 100$ to group 1, covariates $101, \ldots, 200$ to group 2, etcetera. Group G consists of covariates $401, \ldots, 500$. This distribution of covariates among the groups ensures that predictive covariates $\{1, 2, 3\}$ are in Group 1 and that predictive covariates $\{101, 102\}$ are always in Group 2.

We use the following settings for the BART model. The tree hyperparameters are set to $(\alpha = 0.95, \beta = 2, k = 2)$, and we fix the number of trees to $K = 50$. For the error variance hyperparameters, we set $\nu = 10$ and we set λ such that the 75% quantile of the prior equals $2/3\hat{\text{Var}}(\boldsymbol{y})$. For the Gibbs samples, we employ 10 independent chains each containing 10000 samples with a burnin-period of 2000 samples.

For each of the 100 simulated data sets, we run EB-coBART for 40 iterations while tracking the co-data-guided EB estimates of the covariate weights. We show these estimated weights for several iterations in Figure S3 for all simulated data set by depicting boxplots.

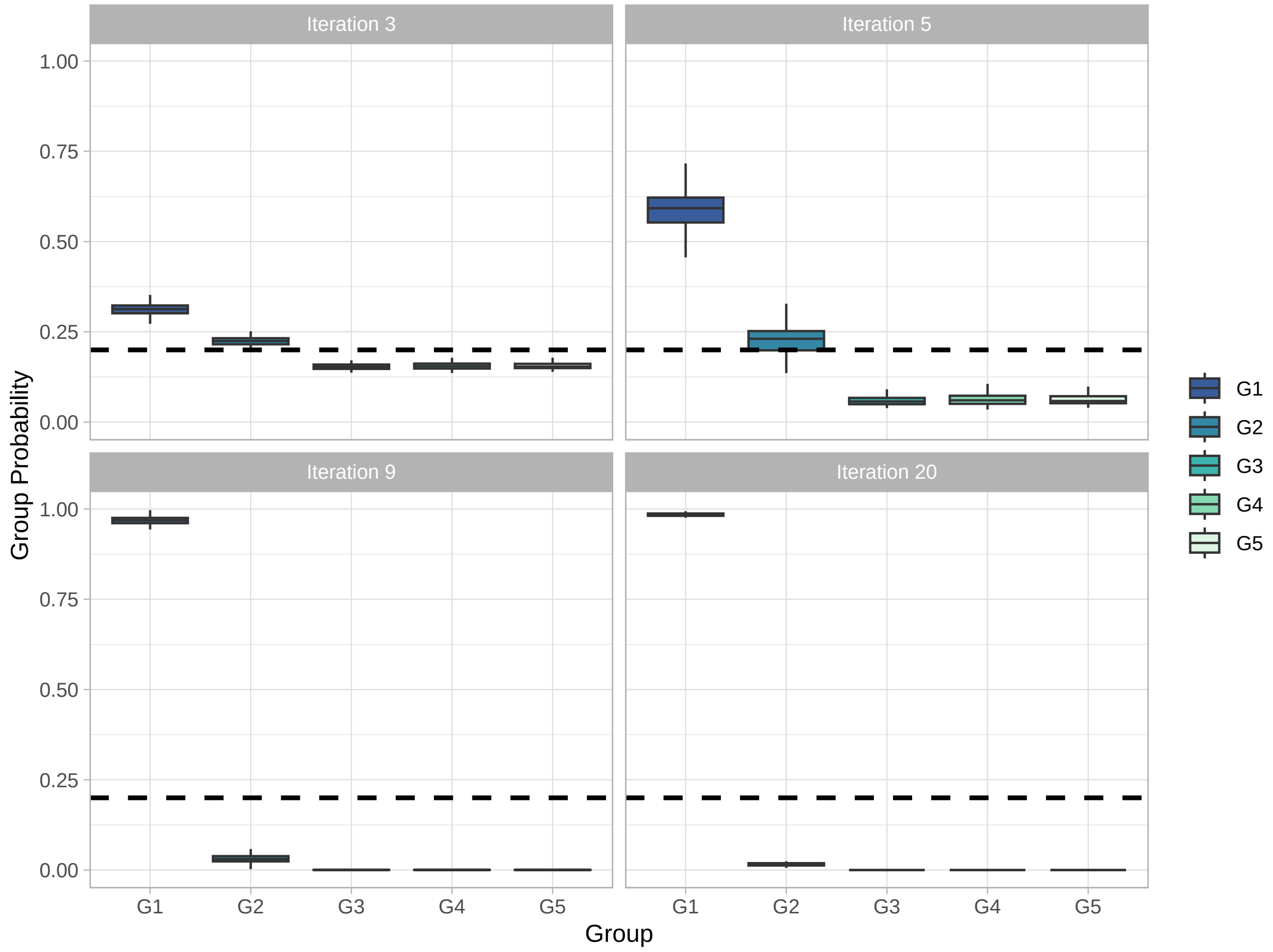

Figure S3: Boxplots of co-data guided EB estimates of the covariate weights across data sets at iteration 3 (top left), 5 (top right), 9 (bottom left), and 20 (bottom right). The vertical dashed lines indicate equal group weights.

Figure S3 illustrates that the estimated group-specific covariate weights converge to the case where almost all probability mass is put on the first group (reached at iteration 15 for all simulated data sets). However, the second group also has predictive covariates and hence it is undesirable to track the co-data-guided EB estimates until these stabilize.

4 Comparing WAIC with cross-validation

Here, we evaluate whether using the WAIC as stopping criterion for the co-data moderated EB updates leads to similar results compared to cross-validation as stopping criterion. To do so, we simulate $N_{sim} = 100$ data sets according to: $Y_i = f_1(\boldsymbol{X}_i) + \epsilon, \quad \text{with} \quad \epsilon \sim \mathcal{N}(0,1), \quad \text{for} \quad i =$

$1, \ldots, N$, and with

$$f_1 = 10 \sin \left(\pi X_{i1} X_{i2}\right) + 20 \left(X_{i101} - 0.5\right)^2 + 10 X_{i3} + 10 X_{i102}, \tag{17}$$

and $X_{ij} \sim \text{Unif}\left(0, 1\right)$, for $j = 1, \ldots, p$ and $p = 500$. Thus, covariates $\{1, 2, 3, 101, 102\}$ are predictive for the response and the remaining 495 covariates are noise.

Co-data is defined as a grouping structure with $G = 5$ groups. We set equal-sized groups of size 100. We then assign covariates $1, 2, \ldots, 100$ to group 1, covariates $101, \ldots, 200$ to group 2, etcetera. Group G consists of covariates $401, \ldots, 500$. This distribution of covariates among the groups ensures that predictive covariates $\{1, 2, 3\}$ are in Group 1 and that predictive covariates $\{101, 102\}$ are always in Group 2.

For each simulated data set, we run EB-coBART while tracking the WAIC (eq. 3.13 of main text), and the cross-validated prediction mean square error (PMSE) utilizing 5 folds. We also compute, at each iteration, the test performance evaluated for $N_{test} = 500$ independent test samples.

We use the following settings for the BART model. The tree hyperparameters are set to $\left(\alpha = 0.95, \beta = 2, k = 2\right)$, and we fix the number of trees to $K = 50$. For the error variance hyper-parameters, we set $\nu = 10$ and we set λ such that the 75% quantile of the prior equals $2/3\hat{\text{Var}}\left(\boldsymbol{y}\right)$. For the Gibbs samples, we employ 10 independent chains each containing 10000 samples with a burnin-period of 2000 samples.

To assess whether WAIC and the cross-validated test performance are comparable as stopping criterion, we compute the ratio of test PMSE at minimum WAIC and at minimum test CV, i.e. $\text{PMSE}_{\text{WAIC}}/\text{PMSE}_{\text{CV}}$, for each of the 100 simulated data sets (Figure S4a). Additionally, we evaluate the difference in estimated group-specific probability between the WAIC and CV as stopping criterion (Figure S4b).

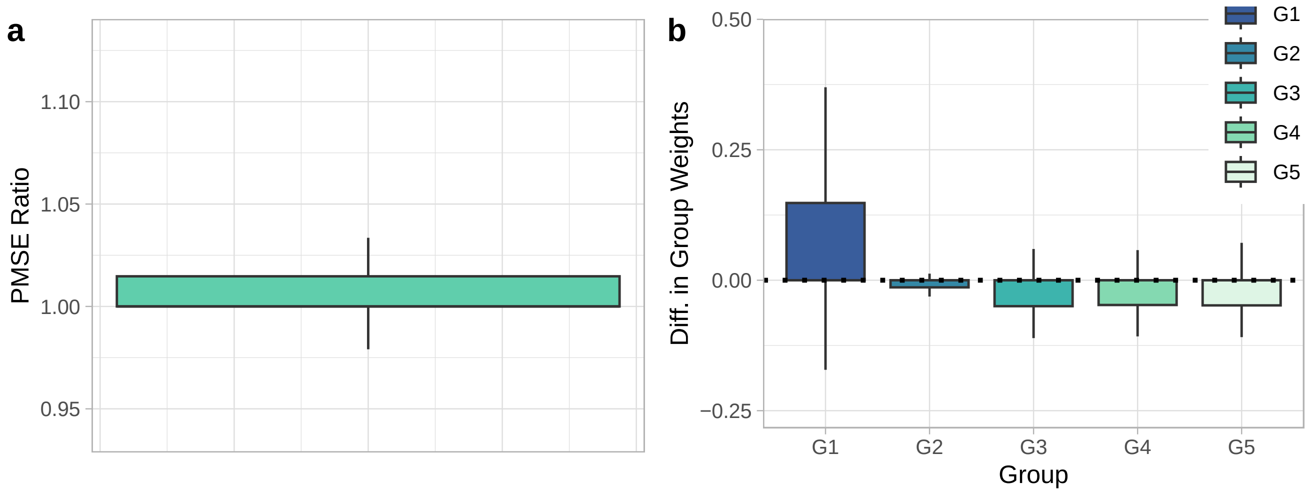

Figure S4: Comparison between WAIC and cross-validation as stopping criterion. **(a)** boxplot of the test performance ratios across the simulated data sets between EB-coBART with the WAIC and EBcoBART with cross-validation as stopping criterion ($\text{PMSE}_{\text{WAIC}}/\text{PMSE}_{\text{CV}}$). **(b)** boxplots of the difference in estimated group probabilities across the data sets between EBcoBART with the WAIC and EBcoBART with cross-validation.

The boxplot of the ratio of the test PMSE at minimum WAIC compared to minimum CV, i.e. $\text{PMSE}_{\text{WAIC}}/\text{PMSE}_{\text{CV}}$, reveals that the predictive performance of the criteria is similar with an average ratio of 1.007 and the difference not larger than 3%. In addition, the difference in estimated group-specific weights does not differ drastically, although for the first group, the difference becomes larger than 0.2 for 13 cases. For roughly half of the cases, the estimated group-specific weights are equal.

5 Variable selection for the sparse and nonlinear simulation setting

We show variable selection results for the sparse and nonlinear simulation setting described in Section 4.1 of the main text. This simulation setting is also described in section 4. To quantify variable selection, we select p_{sel} covariates having the largest count in the splitting rules. For these p_{sel} covariates, we then compute the proportion of predictive covariate counts with respect to the total count of all covariates used in the BART model. The predictive covariates are $j = \{1, 2, 3, 101, 102\}$. We consider $p_{\text{sel}} = \{5, 10, 20\}$. We compute the variable selection statistics for rigid BART, flexible BART, rigid EB-coBART, and flexible EB-coBART (Table S1).

Table S1: Variable selection for BART and EB-coBART in the rigid tree setting ($\alpha = 0.1$, $\beta = 4$, $k = 1$) and the flexible tree setting ($\alpha = 0.95$, $\beta = 2$, $k = 2$). Variable selection is quantified by the proportion of times a predictive covariate is selected in the p_{sel} highest ranked covariates.

	Rigid BART	Rigd EB-coBART	Flexible BART	Flexible EB-coBART
$\boldsymbol{p}_{\textbf{sel}}$	$\boldsymbol{G = 5,\ N = 100}$			
5 best	0.577	0.589	0.091	0.116
10 best	0.580	0.593	0.093	0.117
20 best	0.581	0.594	0.094	0.118
$\boldsymbol{p}_{\text{sel}}$	$\boldsymbol{G = 20,\ N = 100}$			
5 best	0.577	0.730	0.091	0.208
10 best	0.580	0.734	0.093	0.212
20 best	0.581	0.735	0.094	0.214
$\boldsymbol{p}_{\text{sel}}$	$\boldsymbol{G = 5,\ N = 200}$			
5 best	0.818	0.859	0.210	0.241
10 best	0.818	0.860	0.210	0.241
20 best	0.818	0.860	0.210	0.241
$\boldsymbol{p}_{\text{sel}}$	$\boldsymbol{G = 20,\ N = 200}$			
5 best	0.818	0.932	0.210	0.383
10 best	0.818	0.932	0.210	0.383
20 best	0.818	0.932	0.210	0.383

EB-coBART devotes a larger portion of the weight to the predictive covariates compared to BART, i.e. a BART model having equal covariate weights $s_j = 1/p$, in both tree flexibility settings. This effect is stronger for $G = 20$ because the co-data becomes more informative compared to $G = 5$.

Increasing p_{sel} does not yield a larger proportion of selected predictive covariates for $N = 200$. Apparently, selecting $p_{\text{sel}} = 5$, which is also the number of true predictive covariates, is enough. For $N = 100$, there is a (very) small increase in the proportion of true selected covariates.

Rigid BART and rigid EB-coBART have a much larger proportion than their flexible counterparts, as expected. As discussed in the main text, a rigid tree setting favors variable selection.

6 Results for linear and dense simulation setting

Here, we compare the performance of EB-coBART to that of BART, i.e. BART with equal covariate weights $s_j = 1/p$ for a linear covariate-response relationship in a high-dimensional simulation setting.

To specify a dense and linear simulation, we simulate the ith instance of response Y according to $Y_i = \boldsymbol{X}_i \boldsymbol{\theta}^T + \epsilon_i$, with $\epsilon_i \sim \mathcal{N}(0, 1)$, for $i = 1, \dots, N$, and with p-dimensional covariate vector $\boldsymbol{X}_i$ having elements $X_{ij} \overset{i.i.d.}{\sim} \mathcal{N}(0, 1)$, for $j = 1, \dots, p$ and $p = 500$. The p-dimensional regression parameter $\boldsymbol{\theta}$ is a sorted vector, in decreasing order, of values drawn from an $\text{Expo}(1)$. Thus, the first covariate ($j = 1$) is most predictive for the response (largest θ_j value) and the last covariate ($j = 500$) is least predictive. We define continuous co-data by a noised-up version of the true linear effect sizes, i.e. co-data for covariate j equals $\theta_j + \epsilon_j$ with $\epsilon_j \sim \mathcal{N}(0, 0.2\sigma_{\boldsymbol{\theta}})$, for $j = 1, \dots, P$, and with $\sigma_{\boldsymbol{\theta}}$ the standard deviation of $\boldsymbol{\theta}$. We consider two sample sizes: $N = 100$ and $N = 200$, rendering two simulation settings.

Variable importance results are shown in Figure S5. Because EB-coBART estimates feature specific covariate weights for continious co-data, we visualize these estimates by first grouping the covariates in ten groups (first 50 covariates belonging to group 1, the second 50 covariates belonging to group 2, etc.) and then reporting group averages.

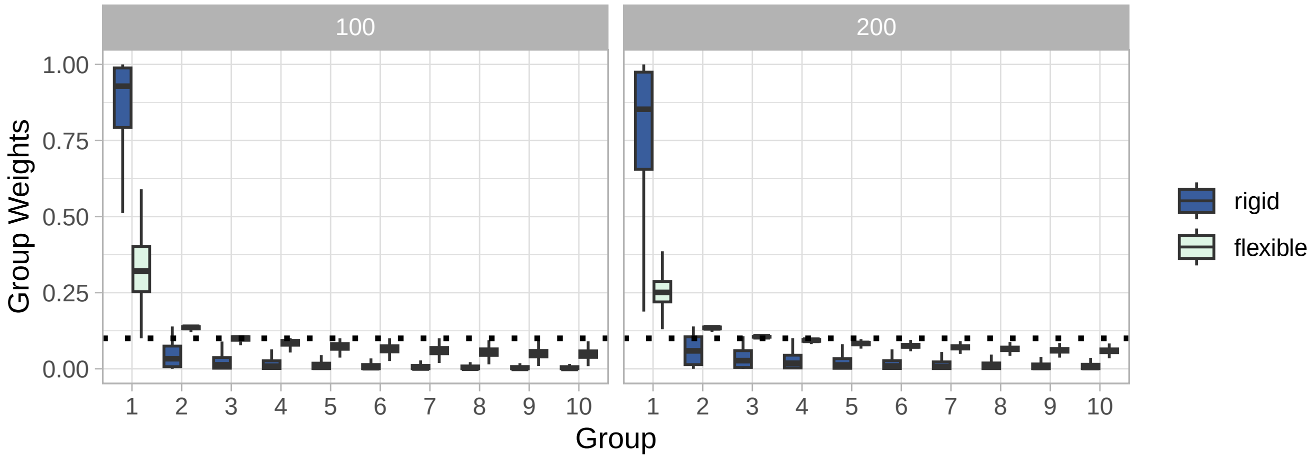

Figure S5: Boxplots of the co-data moderated EB estimates of the covariate weights across the 500 simulated data sets for different simulation settings. Left (blue) boxplots correspond to EB-coBART in the rigid tree setting ($\alpha = 0.1$, $\beta = 4$, $k = 1$) and right (green) boxplots to EB-coBART in the flexible tree setting ($\alpha = 0.95$, $\beta = 2$, $k = 2$). Outliers are not shown. The horizontal dotted lines correspond to equal group weights for $G = 10$.

Figure S5 shows that EB-coBART upweights predictive covariates and downweights less predictive covariates. This up/down-weighting effect is stronger for rigid BART as this model favors variable selection compared to flexible BART. The estimated group weights gradually decrease because groups become less predictive for larger group number.

To compare the predictive performances of EB-coBART and BART, we depict boxplots, across the 500 simulated data sets, of the test PMSE ratios of EB-coBART compared to BART, i.e. $\text{PMSE}_{\text{EBcoBART}}/\text{PMSE}_{\text{BART}}$ for both the rigid tree setting (left, blue) and the flexible tree setting (right, green) (Figure S6). The left panel of Figure S6 corresponds to the $N = 100$ simulation setting, and the right panel corresponds to the $N = 200$ setting. In addition, Table S2 shows the absolute average PMSE for EB-coBART and BART in all tree flexibility and simulation settings. We include a comparison with ridge regression, ecpc (van Nee et al., 2021), random forest, and CoRF (te Beest et al., 2017).

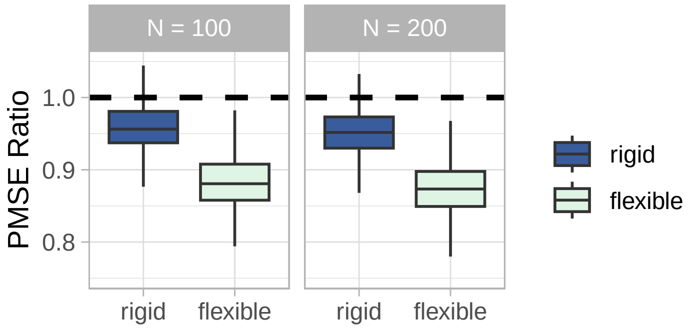

Figure S6: Boxplot of the ratio of the prediction mean square error (PMSE) between BART and EBcoBART ($\text{PMSE}_{\text{BART}}/\text{PMSE}_{\text{EBcoBART}}$) across the 500 simulated data sets for both the rigid tree setting (left, blue) and the flexible tree setting (right, green). The two panels correspond to different simulation settings set by N.

Figure S6 shows that EB-coBART has a smaller PMSE compared to BART for most data sets (indicated by $\text{PMSE}_{\text{EB-coBART}}/\text{PMSE}_{\text{BART}} < 1$). In the rigid tree setting, BART outperforms EB-coBART for 57 data sets in the $N = 100$ simulation setting and 40 data sets in the $N = 200$ setting.

Inspecting the average PMSE values across the 500 simulated data sets reveals that on average EB-BART outperforms BART in both tree flexiblity settings (Table S2). Furthermore, the flexible tree models perform better than the rigid tree models. The linear prediction models ridge regression and ecpc perform much better than the BART models as expected. Ecpc strongly benefits

from the co-data as indicated by the decrease in average PMSE compared to ridge regression.

Flexible EB-coBART has a lower average PMSE than random forest and its co-data extension CoRF. CoRF has a lower PMSE than random forest indicating that CoRF benefits from the co-data. Rigid EB-coBART performs worse than CoRF and slightly better than random forest.

Table S2: Average PMSE across data sets for several simulation settings for BART and EB-coBART in the rigid tree setting ($\alpha = 0.1$, $\beta = 4$, $k = 1$) and the flexible tree setting ($\alpha = 0.95$, $\beta = 2$, $k = 2$). Also included are four competitors: ridge regression, random forest, and co-data learners CoRF and ecpc.

	$N = 100$	$N = 200$
Flexible BART	836.4	787.5
Flexible EB-coBART	744.5	688.6
Rigid BART	883.4	863.0
Rigid EB-coBART	847.8	822.2
DART	836	786
Ridge	733.5	550.2
Ecpc	493.4	241.3
Random forest	857.5	826.6
CoRF	792.4	749.9

7 Uninformative co-data

To define an uninformative co-data setting, we simulate $N_{sim} = 500$ data sets according to: $Y_i = f(\boldsymbol{X}_i) + \epsilon, \quad \text{with} \quad \epsilon \sim \mathcal{N}(0,1), \quad \text{for} \quad i = 1, \ldots, N$, and with

$$\begin{aligned} f = {} & 10\sin(\pi X_{i1}X_{i2}) + 20(X_{i3} - 0.5)^2 + 10X_{i4} + 10X_{i5} \\ & + 10\sin(\pi X_{i101}X_{i102}) + 20(X_{i103} - 0.5)^2 + 10X_{i104} + 10X_{i105} \\ & + 10\sin(\pi X_{i201}X_{i202}) + 20(X_{i203} - 0.5)^2 + 10X_{i204} + 10X_{i205} \\ & + 10\sin(\pi X_{i301}X_{i302}) + 20(X_{i303} - 0.5)^2 + 10X_{i304} + 10X_{i305} \\ & + 10\sin(\pi X_{i401}X_{i402}) + 20(X_{i403} - 0.5)^2 + 10X_{i404} + 10X_{i505} \end{aligned} \tag{18}$$

and $X_{ij} \sim \text{Unif}(0,1)$, for $j = 1, \ldots, p$ and $p = 500$. Sample size N is fixed to $N = 100$

Co-data is defined as a grouping structure with $G = 5$ groups. We set equal-sized groups of size 100. We then assign covariates $1, 2, \ldots, 100$ to group 1, covariates $101, \ldots, 200$ to group 2, etcetera. Group G consists of covariates $401, \ldots, 500$. Thus, each group contributes equally to the overall function ((18)) with the firste five members being predictive and the last 95 members being noise. Therefore, the grouping structure is not informative for the response.

We show the same results as for the simulation in the main text (Subsection 4.1). Figure S7 shows boxplots across the 500 simulated data sets of the group-specific covariate weight estimates $\hat{w}_j^{(q)}$, i.e. eq. 3.12 of the main text, of EB-coBART having a rigid ($\alpha = 0.1$, $\beta = 4$, $k = 1$) or flexible tree setting ($\alpha = 0.95$, $\beta = 2$, $k = 2$). Figure S8 shows the ratio of test performance of EB-coBART compared to BART, i.e. $\text{PMSE}_{\text{EBcoBART}}/\text{PMSE}_{\text{BART}}$ for both the rigid tree setting

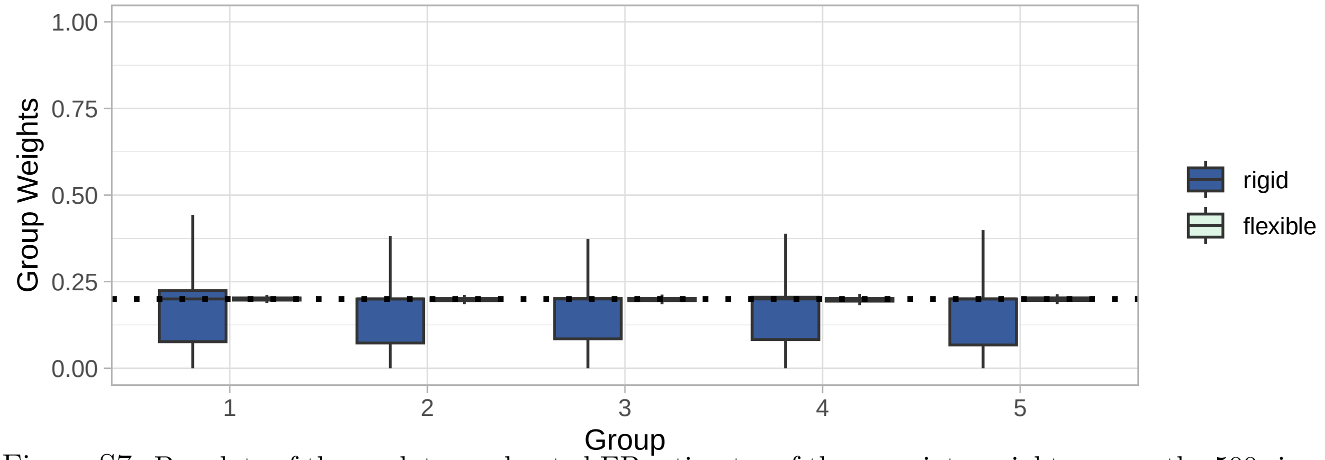

Figure S7: Boxplots of the co-data moderated EB estimates of the covariate weights across the 500 simulated data sets when uninformative co-data is present. For each group, right (blue) boxplot correspond to EB-coBART in the rigid tree setting ($\alpha = 0.1$, $\beta = 4$, $k = 1$) and left (green) boxplot to EB-coBART in the flexible tree setting ($\alpha = 0.95$, $\beta = 2$, $k = 2$). Outliers are not shown. The horizontal dotted line corresponds to equal group weights.

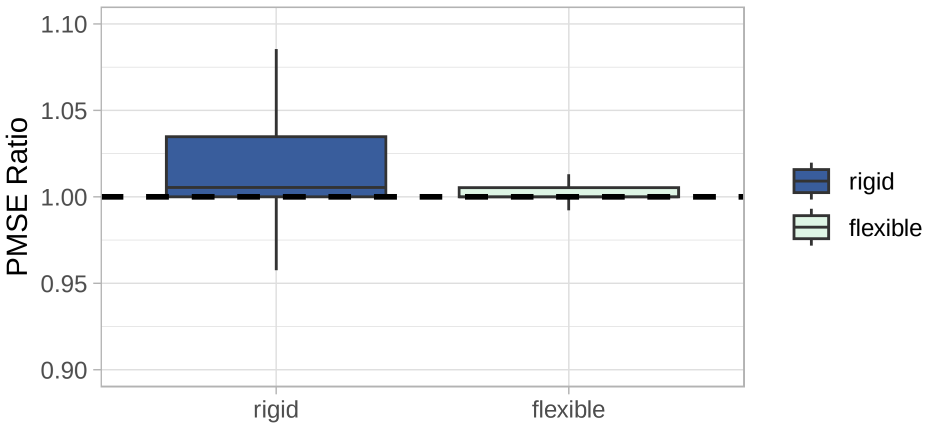

Figure S8: Boxplot of the ratio of the prediction mean square error (PMSE) between BART and EBcoBART ($PMSE_{BART}/PMSE_{EBcoBART}$) across the 500 simulated data sets for both the rigid tree setting (left, blue) and the flexible tree setting (right, green) for uninformative co-data.

The estimated group weights of the rigid tree model (left boxplots) fluctuate around 0.2, i.e. equal group weights (horizontal dotted line). The average and median (accross data sets) of each estimated group weight equal 0.2. The weights of the flexible model (right boxplots) barely fluctuate across the data sets with the average and median also equal to 0.2.

The fluctuations in the estimated weights induce fluctuations in the predictive performance ratio for the rigid tree setting (left boxplot, Figure S8). On average, EB-coBART and BART perform similar, indicated by an average ratio of 1.005. For the flexible tree setting, the PMSE ratio has a relatively constant value of 1.00 across the data sets.

8 Further results of application

Table S3: Predictive performance estimates, based on repeated (3×) 10-fold cross-validation and an external test cohort, of several prediction models.

	**Cross-Validation**		**Test Set Cohort**	
	AUC	Brier score	AUC	Brier score
Rigid BART	0.677	0.166	0.653	0.160
Flexible BART	0.676	0.173	0.556	0.162
cv-BART	0.678	0.168	0.557	0.162
Rigid EB-coBART 1	0.688	0.154	0.692	**0.153**
Rigid EB-coBART 2	0.713	0.155	0.703	**0.153**
Flexible EB-coBART 1	0.715	0.155	0.693	0.154
Flexible EB-coBART 2	0.709	0.156	**0.712**	**0.153**
IPI-BART	0.704	**0.153**	0.669	0.154
DART	0.70	0.171	0.552	0.163
Random Forest	0.688	0.183	0.637	0.158
Corf	0.711	0.157	0.656	0.159
ecpc	**0.728**	0.159	0.701	0.154
Ridge	0.723	0.161	0.689	0.154

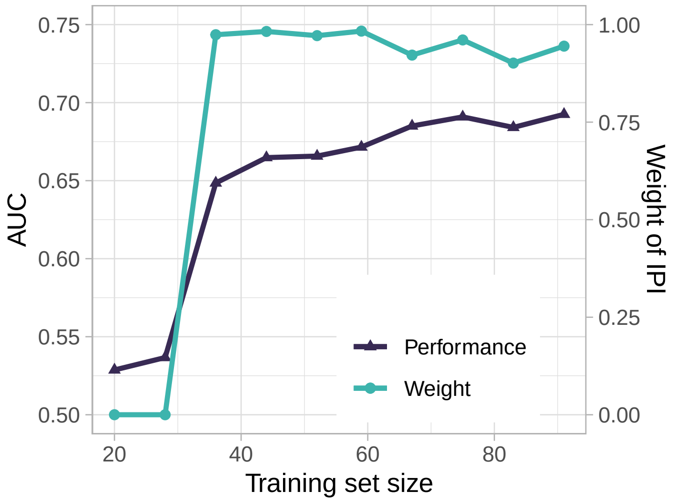

Figure S9: Plot of the AUC (triangles, left y-axis) and the estimated weight of IPI (dots, right y-axis) as a function of the size of the training set.

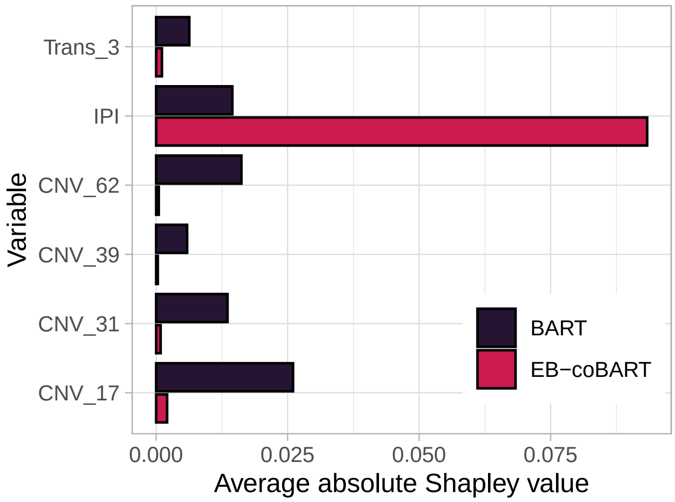

Figure S10: Bar plot of the average absolute Shapley values for BART (black) and EB-coBART (red).

Shapley values are computed using the **fastshap** R package (Štrumbelj and Kononenko, 2014).

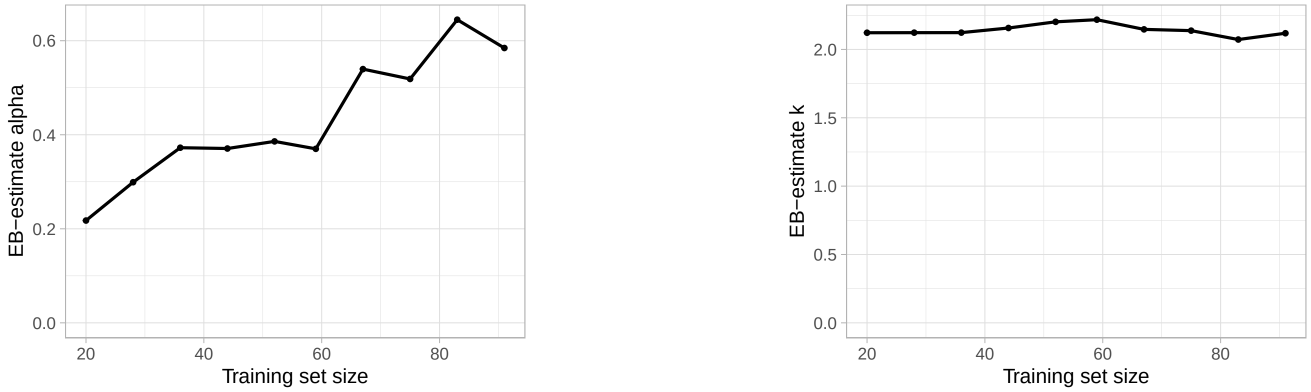

(a) EB-estimates of α for several training set sizes.

(b) EB-estimates of k for several training set sizes.

Figure S11

9 Application 2

This application illustrates the benefit of nonlinear co-data learners compared to linear ones. To do so, we employ the `bloodbrain` dataset from the R package `caret`. This data set has 208 drugs for which the continuous outcome is the concentration ratio in the brain compared to the blood (log scale). The aim is to accurately predict this outcome based on $p = 134$ molecular descriptors such as charge polar surface areas (Mente and Lombardo, 2005).

We split the data set in a co-data set having $N = 166$ samples, on which we estimate a co-data matrix $\boldsymbol{\mathcal{C}}$, and a primary data set having $N = 42$ samples, on which we fit and evaluate the prediction models. We use a relatively small primary data set because the predictive performance of the learners saturates at moderate sample sizes ($N = 60$). At $N = 42$, there is therefore some gain to be expected from the co-data, whereas for larger sample sizes the benefit of co-data will be absent for this application.

From the co-data set, we then estimate ideal co-data for our method EB-coBART, ecpc (van Nee et al., 2021), and CoRF (te Beest et al., 2017) by fitting their corresponding base learners, i.e. BART, ridge regression, and random forest, respectively, and defining the continuous co-data as the estimated weights from these base learners. For EB-coBART and CoRF, these weights correspond to the count of each covariate in the tree ensemble, and for Ecpc, the weights correspond to the estimated effect sizes of the ridge regression.

We then fit and evaluate EB-coBART, Ecpc, and CoRF on the primary data using repeated (3×) 5-fold cross-validation. We fit EB-coBART using 50 trees and $\alpha = 0.95$, $k = 2$, and $\beta = 2$. We also considered the rigid tree setting, i.e. $\alpha = 0.1$, $k = 1$, and $\beta = 4$, but this setting performed worse. We fix the error variance, i.e. σ^2, hyperparameters $\nu = 10$ and λ such that the 75% quantile of the prior equals $2/3\hat{\text{Var}}\left(\boldsymbol{y}\right)$, with $\hat{\text{Var}}\left(\boldsymbol{y}\right)$ the estimated variance of the response $\boldsymbol{y}$. BART is fitted using 10 independent MCMC chains each consisting of 40000 samples of which half is burn-in. Random forest is fitted using 200 trees. For ECPC, we also performed the post-hoc variable selection procedure explained in the main text, but this did not improve performance.

Predictive performance results, quantified by the prediction mean square error (PMSE) and R^2, are shown in Table . For completeness, we also show the performances of the corresponding base learners.

Table S4: Average PMSE across data sets for several simulation settings for BART and EB-coBART in the rigid tree setting ($\alpha = 0.1$, $\beta = 4$, $k = 1$) and the flexible tree setting ($\alpha = 0.95$, $\beta = 2$, $k = 2$). Also included are four competitors: ridge regression, random forest, and co-data learners CoRF and ecpc.

	PMSE	R^2
BART	0.420	0.375
EB-coBART	0.378	0.438
Ridge	0.590	0.103
Ecpc	0.508	0.239
Random forest	0.404	0.406
CoRF	**0.357**	**0.471**

The tree-based co-data learners, which are nonlinear, have a smaller PMSE (and larger R^2) than regression-based Ecpc. CoRF performs slightly better than EB-coBART. All co-data learners show a benefit from the co-data with respect to their corresponding base-learners.

10 Sofware and data availability

R code to reproduce results is available via `https://github.com/JeroenGoedhart/EB_coBART_paper`. We use R version 4.3.0. The repository EB-coBART has an Application map, where the anonymized data and scripts to reproduce results presented in the application are located, and a Simulation map, which contains R scripts to reproduce the simulation results. Seeds for the pseudo random number generator are found in the scripts.

The anonymized data is a list object with 5 elements: the train covariate data, the train response, the test covariate data, the test response, and the co-data matrix, which consist of the external information on the covariates: a grouping by covariate type and p-values on the $-$logit scale.

We used the following R packages throughout the article: dbarts (version $0.9-23$) to fit BART models, BART (version 2.9.6) to fit DART, loo (version 2.6.0) to estimate the WAIC, ecpc (version 3.1.1) to fit ecpc and ridge regression, randomForestSRC (version 3.2.2) to fit random forest and CoRF, pROC (version 1.18.4) to estimate the AUC, ggplot2 (version 3.4.2) to plot

results.

References

T. Hastie and R. Tibshirani. Bayesian backfitting. *Stat Sci*, 15(3):196–223, 2000. doi: 10.1214/ss/1009212815.

Y. V. Tan and J. Roy. Bayesian additive regression trees and the general bart model. *Stad Med*, 38(25):5048–5069, 2019. doi: 10.1002/sim.8347.

A. Kapelner and J. Bleich. bartmachine: Machine learning with bayesian additive regression trees. *J Stat Softw*, 70(4):1–40, 2016. doi: 10.18637/jss.v070.i04.

G. Casella. Empirical Bayes Gibbs sampling. *Biostatistics*, 2(4):485–500, 2001. doi: 10.1093/biostatistics/2.4.485. URL https://doi.org/10.1093/biostatistics/2.4.485.

M. M. van Nee, L. F. A. Wessels, and M. A. van de Wiel. Flexible co-data learning for high-dimensional prediction. *Stat Med*, 40(26):5910–5925, 2021. doi: https://doi.org/10.1002/sim.9162. URL https://doi.org/10.1002/sim.9162.

D. E. te Beest, S. W. Mes, S. M. Wilting, R. H. Brakenhoff, and M. A. van de Wiel. Improved high-dimensional prediction with random forests by the use of co-data. *BMC Bioinformatics*, 18(1):584, 2017. doi: 10.1186/s12859-017-1993-1. URL https://doi.org/10.1186/s12859-017-1993-1.

E. Štrumbelj and I. Kononenko. Explaining prediction models and individual predictions with feature contributions. *Knowl Inf Syst*, 41(3):647–665, Dec 2014. doi: 10.1007/s10115-013-0679-x. URL https://doi.org/10.1007/s10115-013-0679-x.

S. R. Mente and F. Lombardo. A recursive-partitioning model for blood–brain barrier permeation. *J Comput Aided Mol Des*, 19(7):465–481, Jul 2005. doi: 10.1007/s10822-005-9001-7. URL https://doi.org/10.1007/s10822-005-9001-7.
